# Quantitative Description of Intrinsically Disordered Proteins Using Single-Molecule FRET, NMR, and SAXS

**DOI:** 10.1021/jacs.1c06264

**Published:** 2021-11-24

**Authors:** Samuel Naudi-Fabra, Maud Tengo, Malene Ringkjøbing Jensen, Martin Blackledge, Sigrid Milles

**Affiliations:** Institut de Biologie Structurale, Université Grenoble Alpes-CEA-CNRS, 71, Avenue des Martyrs, 38044 Grenoble, France

## Abstract

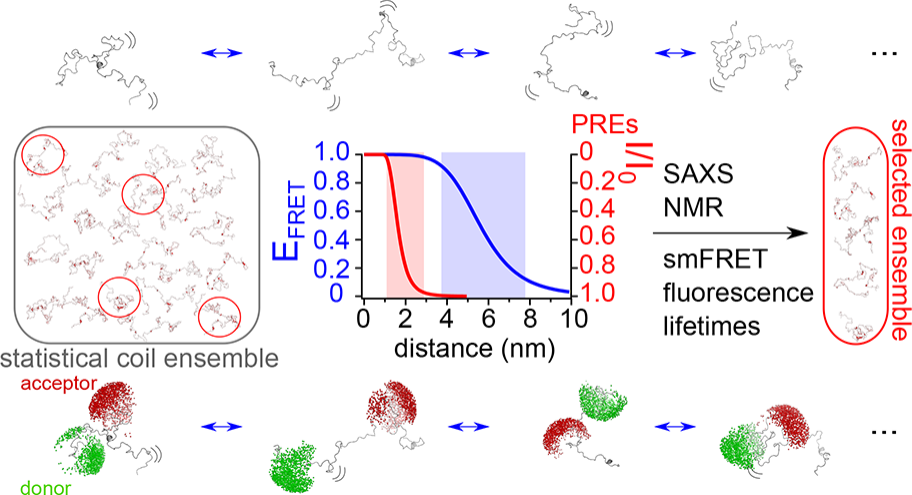

Studying the conformational landscape of intrinsically disordered and partially folded proteins is challenging and only accessible to a few solution state techniques, such as nuclear magnetic resonance (NMR), small-angle scattering techniques, and single-molecule Förster resonance energy transfer (smFRET). While each of the techniques is sensitive to different properties of the disordered chain, such as local structural propensities, overall dimension, or intermediate- and long-range contacts, conformational ensembles describing intrinsically disordered proteins (IDPs) accurately should ideally respect all of these properties. Here we develop an integrated approach using a large set of FRET efficiencies and fluorescence lifetimes, NMR chemical shifts, and paramagnetic relaxation enhancements (PREs), as well as small-angle X-ray scattering (SAXS) to derive quantitative conformational ensembles in agreement with all parameters. Our approach is tested using simulated data (five sets of PREs and 15 FRET efficiencies) and validated experimentally on the example of the disordered domain of measles virus phosphoprotein, providing new insights into the conformational landscape of this viral protein that comprises transient structural elements and is more compact than an unfolded chain throughout its length. Rigorous cross-validation using FRET efficiencies, fluorescence lifetimes, and SAXS demonstrates the predictive nature of the calculated conformational ensembles and underlines the potential of this strategy in integrative dynamic structural biology.

## Introduction

Intrinsically disordered proteins (IDPs) play important roles in many biological systems and exert their tasks thanks to their ability to sample conformational ensembles that can have different degrees of compactness and that often comprise transiently folded regions functioning as interaction sites.^1,2^ Although IDPs are known to be devoid of stable secondary and tertiary structures, primary structure determines their function and modulates the conformations sampled on a rapid time scale: small motifs can locally enrich the IDP in hydrophobic amino acids, and clusters of charged residues may lead to self-repulsion, thus affecting the properties of the chain.^3−5^

Single-molecule Förster resonance energy transfer (smFRET) has demonstrated to be a very powerful tool to access the dimension of the unfolded chain through the measurement of energy transfer between site-specifically attached donor and acceptor fluorophores as a function of their distance.^6,7^ The technique is compatible with very large IDPs,^8^ covering distances that range from 2 to 10 nm approximately, and structural information can be obtained in the presence of transiently folded or folded domains,^9^ in complex environments, and even within the living cell.^10,11^ Obtaining quantitative structural insight has, however, remained challenging in particular as the distance between the fluorophores, rather than between their attachment points in the protein backbone, is determined experimentally, and the chemical composition of the dyes and their linkers therefore has to be taken into account in structural modeling. For folded proteins, recent advances have overcome this problem by generating structural models explicitly considering the attached fluorophores mainly through calculation of the volumes that the fluorophores can occupy when attached to a specific site in the protein (accessible volumes, AVs).^12−15^

Determination of distances for IDPs suffers from the additional challenge that the measured FRET efficiency (*E*_FRET_) describes an ensemble of distances rather than an individual distance, which has frequently been taken into account by assuming the sampling of a Gaussian chain (or other polymer-) distribution between the fluorophores.^16^ These distributions can be expressed as a function of the number of amino acids between the attachment points of the fluorophores, and in order to consider the contribution of the fluorophores and their linkers to the measured distance, they are usually assumed to contribute a number of additional residues. Although this approach has led to distance distributions in agreement with conformational ensembles derived from other experimental techniques (nuclear magnetic resonance, NMR, and small-angle X-ray scattering, SAXS),^17^ the number of amino acids that has to be added to consider the dyes and their linkers is not unambiguous.^17−19^ This has consequences when distances within IDPs are measured by different techniques. Radii of gyration (*R*_G_) measured using SAXS and those inferred from end-to-end distances (*R*_E_) using smFRET have apparently disagreed for a long time.^20−22^ A number of approaches have been presented to resolve this controversy, employing improved analysis procedures and explicit ensembles, generated using Bayesian statistics or maximum entropy approaches, in agreement with smFRET and SAXS.^19,23−25^ In this context, fluorophores have been attached *in silico* to describe measured *E*_FRET_ of individual distances (one distance per protein).^19,26^ While these approaches are promising, the study of IDPs demands a systematic analysis integrating distance information between different regions of the protein, its global extension, but also local structural information to accommodate heterogeneity in compaction, as well as population of transiently structured elements.

Here, we propose an approach for the systematic integration of various solution state structural data of IDPs based on the implementation of FRET efficiencies into the algorithm ASTEROIDS that derives representative structural ensembles of IDPs from NMR and SAXS data describing both local conformational propensities and long-range distance information.^27,28^ Our approach is based on the selection of smaller ensembles from a large statistical coil ensemble (calculated using flexible-meccano^29^ and of an extension approximately equal to a fully unfolded protein^30^) solely using experimental data, and the fluorophores are explicitly taken into account through the per-conformer calculation of AVs. This strategy does not require a conversion between different distance measures (e.g., *R*_G_ and *R*_E_), nor does it require an approximation of the dyes/linker length in the context of a polymer model and therefore allows describing IDPs of varying degrees of compactness along their sequence, theoretically even including entirely folded domains. We first selected and cross-validated conformational ensembles using a large set of *in silico* PRE (paramagnetic relaxation enhancement) and FRET data. Finally, we validate our approach with respect to experimental FRET efficiencies, SAXS data, as well as NMR chemical shifts and PREs, obtaining new insights into the conformational landscape of an intrinsically disordered region of the measles virus phosphoprotein. Notably, in addition to a number of FRET efficiencies and SAXS data, we also use experimental fluorescence lifetimes of the FRET-labeled protein to cross-validate our conformational ensemble, demonstrating correct sampling of the ensemble itself as well as the dye AVs. We demonstrate complementarity between different parameters (particularly FRET and PREs) and the importance of using distance information across the IDP sequence to generate meaningful conformational ensembles. The presented approach now allows addressing dynamic integrated structural biology quantitatively and in a predictive manner.

## Results

### FRET Distance Networks in Conformational Ensembles

In order to determine conformational ensembles based on experimental smFRET data, we build on an approach that has been developed and frequently used for calculating conformational ensembles based on diverse NMR parameters and SAXS.^1,31−34^ A large ensemble of conformers (e.g., 10 000) is calculated based on a statistical distribution of Φ and Ψ angles of the protein backbone using the software flexible-meccano.^29^ From this large ensemble, smaller subensembles that describe the experimental data are selected using the genetic algorithm ASTEROIDS.^35^

Distance measurements through FRET rely on the attachment of a donor and an acceptor fluorophore to specific sites within the protein chain. Our goal being to describe the experimental FRET efficiencies directly, the fluorescent dyes thus have to be accounted for in the conformational ensemble. We calculated accessible volumes for the fluorophores Alexa488 and Alexa594 attached to cysteines via maleimide chemistry and comprising a C_5_ linker connecting the Cys side chain and the fluorophore, as previously described.^12,36^ As a first step, we calculated a conformational ensemble of a 110 amino acid long model protein containing two cysteines as dye attachment points, and we calculated AVs for every conformer in the ensemble. Both sampling of the AV and sampling of the different conformers were assumed to be on a time scale significantly slower than the fluorescence lifetime, as suggested by AV sampling based on molecular dynamics simulations of fluorescently labeled DNA.^37^ We first used the cysteine side chains as attachment points. In order to allow labeling positions that are not native cysteines and that are experimentally generated through point mutations, we estimated the average distance between the C_β_ atom and the SH and elongated the linker length in the simulation accordingly (see Methods). The distance distributions calculated on a 100 conformer ensemble with attachment points at either the SH or C_β_ with their respective parametrization of the linker can be considered equal (SI Figure 1).

**Figure 1:**
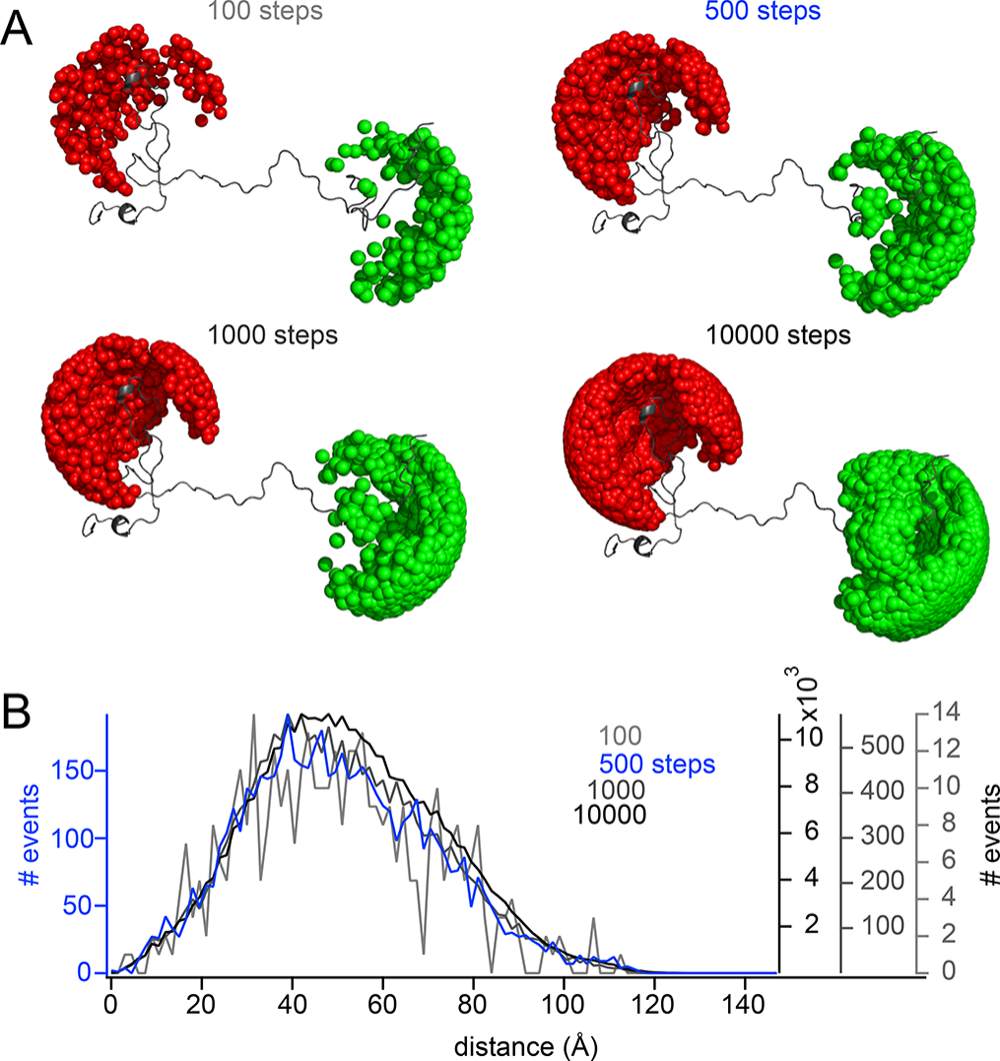
Influence of step size on conformational ensembles. (A) Examples of conformations of a model protein with accessible volumes (AVs) of Alexa488 (green) and Alexa594 (red), calculated using 100, 500, 1000, or 10 000 iterations (steps) for position determination. (B) Distance histogram over accessible volumes calculated over a 100 conformer ensemble using 100 (light gray), 500 (blue), 1000 (dark gray), and 10 000 (black) dye positions sampled iteratively.

For the selection of meaningful ensembles, AVs have to be calculated and FRET efficiencies determined for all conformers in the large flexible-meccano ensemble before selection using ASTEROIDS. Since AV calculation is time-consuming, the iterative sampling of positions in the AV was optimized to 500 iterations (Figure 1) and the pairwise distance calculation coarsened (see Materials and Methods).

### Benchmarking an Ensemble Selection Using FRET against *in Silico* Data

After optimizing AV calculations for multiconformational ensembles, we investigated whether FRET efficiencies (*E*_FRET_) could be used in the context of the ensemble selection algorithm ASTEROIDS. For this, we used an IDP sequence of 155 amino acids in length, for which we calculated an ensemble comprising a long-range contact between amino acid segments 15–25 and 90–100 and for which we generated 15 *in silicoE*_FRET_ (SI Figure 2) using AV calculations as described above. In order to obtain distances that adequately reflect the long-range behavior of the ensemble, we selected labeling positions covering different regions of the protein and care was taken to cover both short and long amino acid distances between the attachment points of the labels so as to address FRET efficiencies throughout the sensitive regime of FRET (around 2–10 nm).

**Figure 2:**
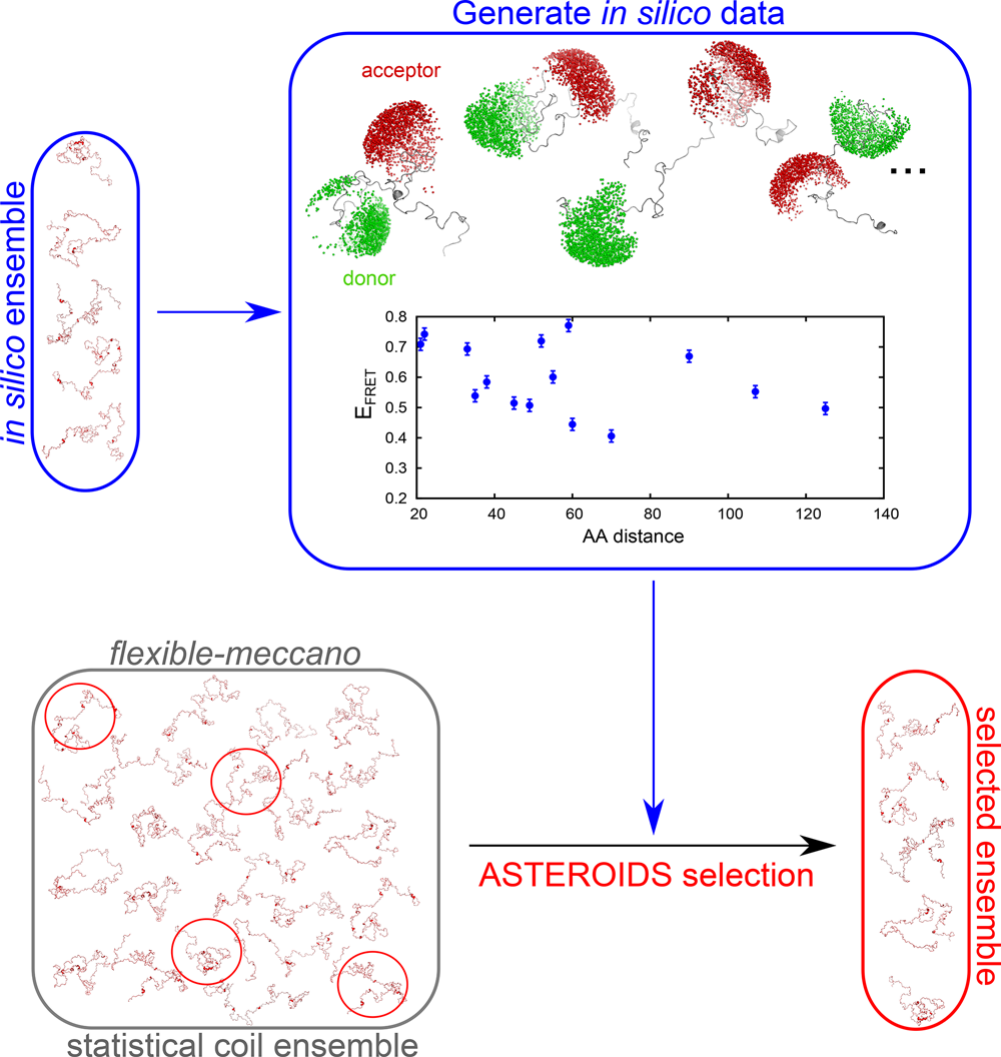
Scheme of incorporation of FRET distances into ASTEROIDS based on simulated data. An *in silico* ensemble of conformations is generated, for which accessible volumes occupied by Alexa488 and Alexa594 are computed and FRET data are calculated (blue frame). FRET efficiencies are used as an input for ASTEROIDS selection (red frame) from a pool of statistical coil conformers (calculated from flexible-meccano, gray frame).

From a large statistical-coil ensemble calculated using flexible-meccano, we then selected smaller subensembles of 200 conformers in size using ASTEROIDS based on six of the 15 *in silico* FRET efficiencies (Figure 2). When the remaining nine FRET efficiencies, that were not used in the selection, were back-calculated from the selected ASTEROIDS ensemble, the *in silico* FRET efficiencies of the input ensemble comprising a long-range contact were predicted with high accuracy (SI Figure 3A).

The FRET efficiencies used in this selection were chosen to represent varying distances across the sequence of the protein, and sufficient sampling of the different regions of the protein is indeed crucial for reproducing the long-range characteristics of the ensemble with confidence. If only three FRET efficiencies were used in the selection, even when distributed along the sequence, the remaining FRET efficiencies not used in the selection were only poorly predicted by the ASTEROIDS ensemble and the long-range distances of the simulated target ensemble much less well captured (SI Figure 4).

### PREs and FRET Distances Provide Complementary Long-Range Distance Information

**Figure 3:**
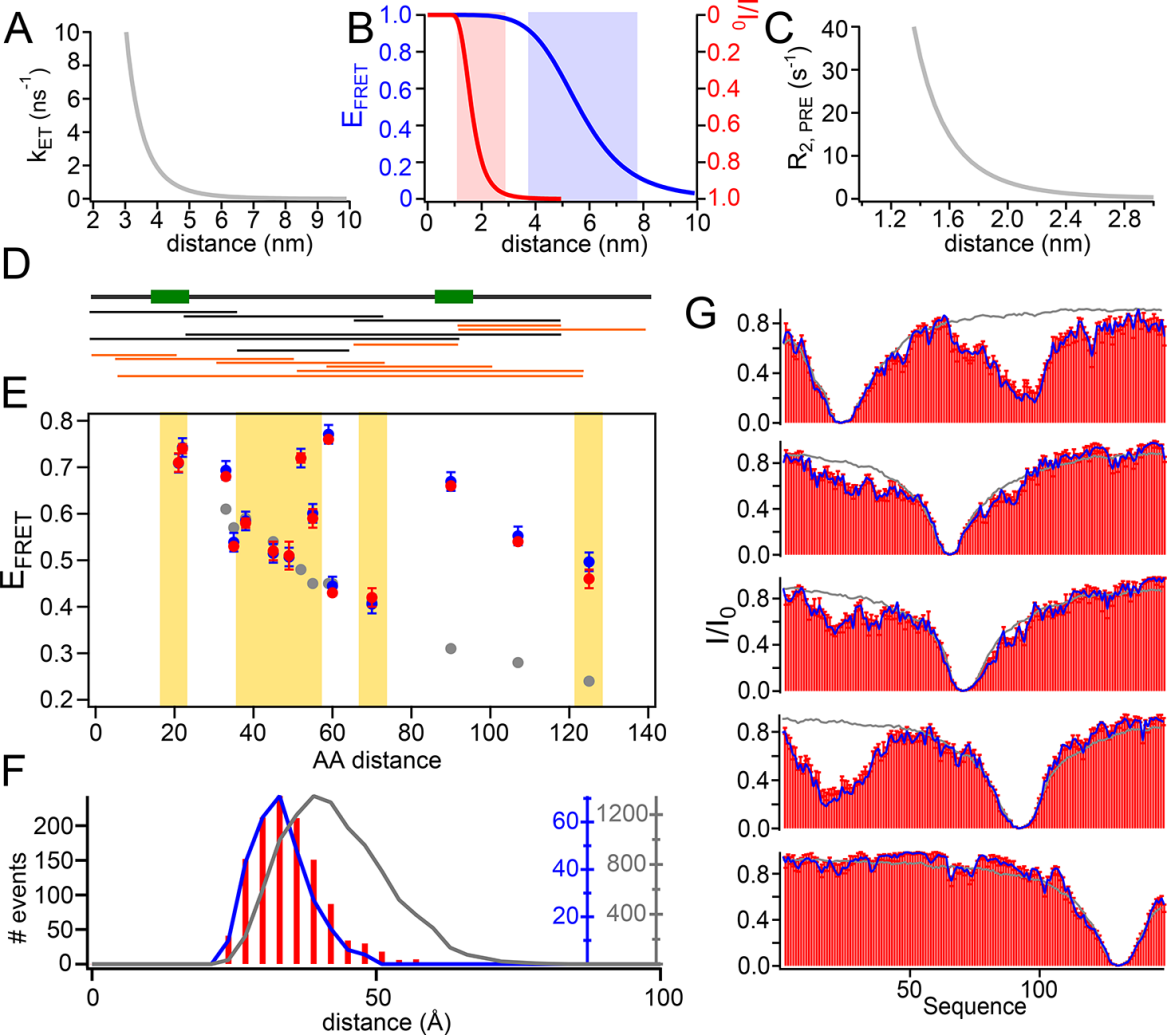
ASTEROIDS selection based on PREs and FRET efficiencies. (A) Dependence of the FRET rate (*k*_ET_) on distance with a Förster radius of 56 Å and a fluorescence lifetime of the donor (τ_D_) of 4 ns. (B) Dependence of the FRET efficiency (*E*_FRET_) on distance for a *k*_ET_ as displayed in A (blue curve, blue *y* axis). The red curve with the red *y* axis shows the dependence of peak intensity ratios (*I*/*I*_0_) for a paramagnetic as compared to a diamagnetic sample at a PRE rate described in C. Red and blue shading illustrate the distance ranges to which FRET and PREs are sensitive. (C) PRE rate (*R*_2,PRE_) dependence on the distance between the proton and electron spins for τ_c_ = 5 ns, with τ_c_ = τ_r_τ_s_/(τ_r_ + τ_s_), τ_r_ being the rotational correlation time of the protein and τ_s_ the effective electron relaxation time (see ref (38)). Note that reorientation dynamics of the spin label was not taken into account for this illustration, but was considered in the ensemble calculations. (D) Schematic of a 155 residue long IDP comprising a long-range interaction between regions indicated by green boxes. Below: Distances for which *E*_FRET_ has been calculated after *in silico* addition of the fluorescent dyes. Black protein constructs have been used in the ASTEROIDS selection; orange protein constructs have been used for cross validation (corresponding *E*_FRET_ above yellow background in E). See also SI Figure 2 for a more detailed scheme. (E) FRET efficiencies plotted against the amino acid distance between the labels of a flexible-meccano ensemble (gray), the simulated ensemble with a long-range contact (blue), and an ensemble selected based on six FRET efficiencies and five PRE labeling positions (red). Only *E*_FRET_ on a white background have been used in the selection. Cross-validated distances are on a yellow background. Error bars on the blue points indicate the error in FRET efficiency that was allowed in the selection (0.02). Error bars on the red points refer to the standard deviation of *E*_FRET_ calculated from six independent selections. (F) Histogram of average pairwise C_α_–C_α_ distances of the flexible-meccano ensemble (gray), the simulated ensemble (blue), and the ensemble selected based on PREs and six different FRET distances (red bars). (G) PREs of a flexible-meccano ensemble (gray lines), of the simulated ensemble with a long-range contact (blue lines), and of the selected ensemble (red bars). All simulated PREs (in blue) were used in the selection. Red error bars are standard deviations over six independent selections.

Through paramagnetic relaxation enhancements, NMR also offers a probe for longer range distances that can reach up to around 2.5 nm.^38^ For this, a paramagnetic probe (usually a spin radical) is attached to a site-specifically engineered cysteine within the protein chain, and its effects on spin relaxation of the different ^1^H_N_ nuclei within the protein backbone are measured and depend on the inverse sixth order of the respective distance from the spin radical. PREs thus have a distance dependence similar to FRET with, however, different sensitive regimes (Figure 3A–C). Indeed, the distance windows at which FRET and PREs are sensitive, respectively, are entirely complementary, and only both techniques together are expected to provide insights into both intermediate (around 1–3 nm) and long-range (around 4–8 nm) distance ranges.

While the contribution of paramagnetic relaxation can be directly determined through the measurement of spin relaxation, we do not have access to the FRET rate (*k*_ET_) itself, which can only be measured indirectly through the fluorescence lifetime of the donor in the absence (τ_D_) and presence (τ_D(FRET)_) of the acceptor,
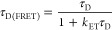
1or through the FRET efficiency,
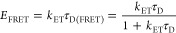
2leading to a dampened dependence between the measurement parameter (τ_D_ or *E*_FRET_) and the donor–acceptor distance for short distances. A similar dependence can be obtained if peak intensity ratios (*I*/*I*_0_) of the para- and diamagnetic PRE sample are considered, allowing a visual inspection of the complementary distance ranges (Figure 3B). We calculated PREs^39^ using five different attachment sites for a spin radical in our long-range ensemble and used these *in silico* PREs to select smaller subensembles of 200 conformers using ASTEROIDS. Although all PREs are captured very well in these ensembles, they fail to reproduce the expected FRET efficiencies (SI Figure 3C,D). This observation remains true also if fast (faster than the fluorescence lifetime) sampling of the AVs was assumed (SI Figure 5). The selection based on six FRET efficiencies described above, on the other hand, also fails to reproduce the expected PREs, thus illustrating the expected complementary distance ranges to which PREs and FRET are sensitive (SI Figure 3B).

An ensemble that has been selected based on five *in silico* PRE labeling sites and six FRET efficiencies, however, leads to an excellent reproduction of all *in silico* PREs and *E*_FRET_ (Figure 3), and this ensemble also reliably reproduces the expected average pairwise as well as specific C_α_–C_α_ distance distributions (Figure 3F, SI Figure 6) that can be calculated directly from the selected ensemble without additional approximation concerning fluorescent dyes and their linkers (or PRE labels).

Indeed FRET efficiencies and PREs are both necessary to correctly describe a conformational ensemble that populates various intermediate- and long-range distances. Including only FRET or only PREs into a selection can only be expected to reproduce the respective other parameter for a very narrow distance window and depending on the properties of the pool of conformers from which ensembles are selected. We demonstrate this on the example of a new set of *in silico* data, in which we allowed the long-range contact to reach up to 50 rather than 20 Å, to which FRET efficiencies, but not PREs are sensitive. In this case, selection based on six FRET efficiencies leads to agreement with the *in silico* PREs, which are not noticeably different from a flexible-meccano-derived statistical coil (SI Figure 7).

### Analysis of Ensemble Sizes

**Figure 4:**
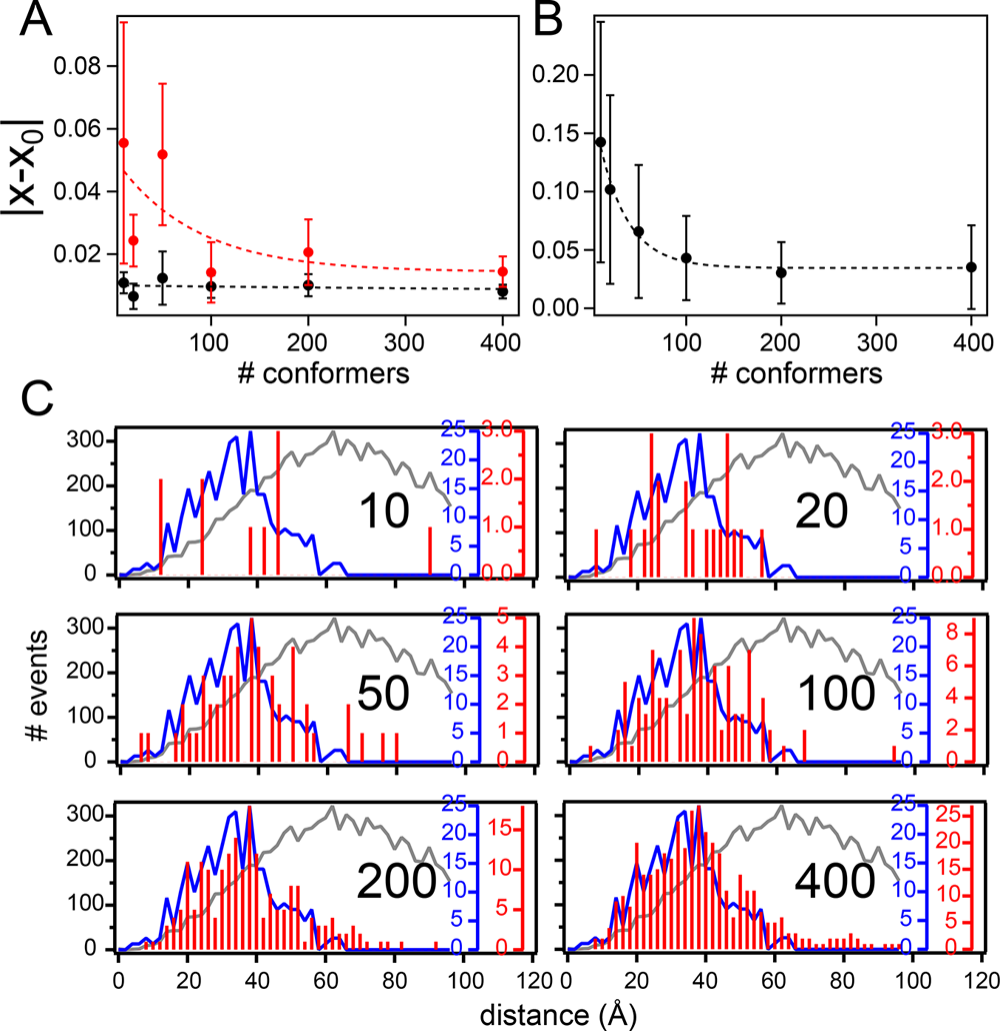
Varying the size of the selected ensemble. (A and B) Averaged absolute deviations of the FRET efficiency (A) or PRE (B) as calculated from the selected ensemble (*x*) from the respective values of the target *in silico* ensemble (*x*_0_). Error bars show the corresponding standard deviations. Red points illustrate data not used in the selection. Ensemble sizes were 10, 20, 50, 100, 200, or 400 conformers. Dashed lines represent exponential fits representing the trend of the data. (C) C_α_–C_α_ distances between the *in silico* labeling sites 2 and 92 for different ensemble sizes. In red are the distances calculated from one selection based on six FRET efficiencies and using five PRE labeling sites. The expected C_α_–C_α_ distances are shown in blue; the distances obtained from a flexible-meccano statistical coil ensemble in gray. Black numbers inside the graphs indicate the numbers of conformers used in the selected ensembles.

Ensemble selections based on *in silico* data back-calculated from a known target ensemble also allowed us to test the number of conformers required to represent the data and sufficient to reliably reproduce the statistics of the target ensemble. We have thus performed selections of 10, 20, 50, 100, 200, and 400 conformers per ensemble and calculated average absolute deviations from the *in silico* data. This analysis indicates that reproduction of the data improves as the ensemble size increases (Figure 4A and B), reaching excellent agreement with the *in silico* data starting from around 200 conformers per ensemble. Reproduction of the C_α_–C_α_ distance distributions between the labeling sites is comparatively poor at low numbers of conformers, and only starts improving once an ensemble size of approximately 100 conformers is reached. Reproduction further improves with increasing numbers of conformers (Figure 4C and SI Figure 6). We thus conclude that, overall, an ensemble size of 200 conformers, as proposed earlier for ensembles selected based on PREs and residual dipolar couplings,^39^ is a good size to reconcile reproducibility, statistics, and computation speed.

### Description of Experimental FRET, PREs, and Chemical Shift Data

**Figure 5:**
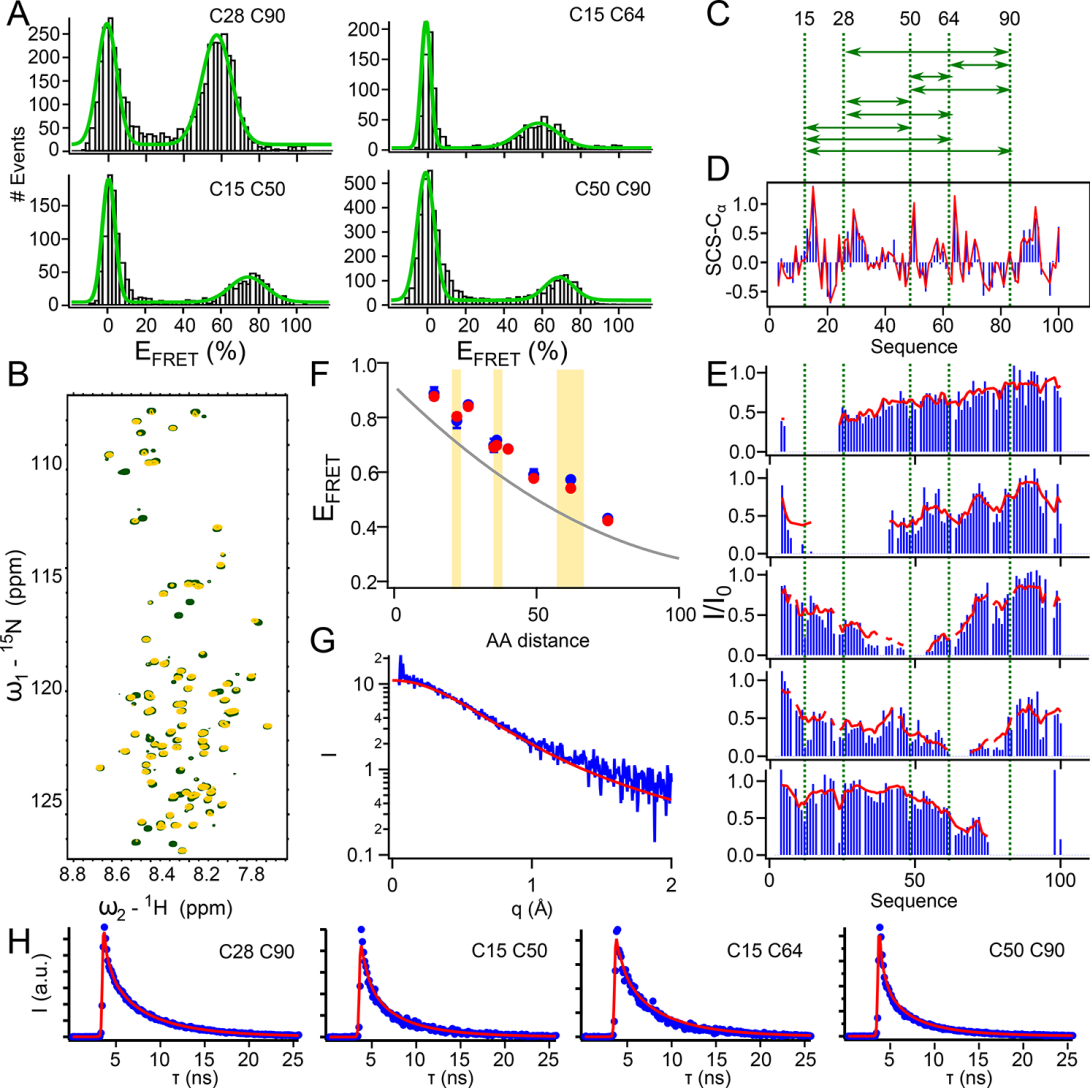
Description of experimental FRET, PREs, and SAXS by a common multiconformational model. (A) Experimental FRET histograms of P_1–100_ (black bars) with double Gaussian fit (green) from which *E*_FRET_ of the nonzero population was extracted. (B) ^1^H–^15^N heteronuclear single quantum coherence (HSQC) spectrum of P_1–100_ C64 unlabeled (green) and labeled with MTSL (yellow). (C) Visualization of FRET distances for which data have been acquired. (D) C_α_ secondary chemical shifts of P_1–100_ calculated based on experimental chemical shifts (blue) and based on chemical shifts calculated from an ensemble selected based on five PRE labeling positions, six FRET efficiencies and chemical shifts (red). (E) Experimental (blue) PREs and PREs calculated from the selected ensemble (red). All PREs were used in the selection. PRE labeling sites are indicated by green dashed lines (note that the same cysteines have been used for PRE and FRET labeling). Intensity ratios between the PRE labeled (*I*) and unlabeled (*I*_0_) peaks are shown. (F) FRET efficiencies (*E*_FRET_) of P_1–100_ plotted against the amino acid distance between the fluorophores. The gray line indicates values expected from a flexible-meccano statistical coil (polynomial fit of *in silico* data presented in Figure 3). Experimental data are shown in blue with error bars resulting from standard deviations calculated from independent measurements. Red points indicate *E*_FRET_ calculated from the ASTEROIDS selection. Data points plotted in front of a yellow background were not used in the selection. (G) Experimental SAXS curve (blue) and SAXS curve back-calculated from the ASTEROIDS ensemble (red). SAXS data were not used in the selection. (H) Cumulated fluorescence lifetime histograms calculated from the FRET population of the single molecule data (corresponding to FRET mutants shown in (A)). Blue points are experimental data, and red curves are decays back-calculated from the selected ensemble, comprising a scattering contribution and scaled to best fit the experimental data.

While our comprehensive *in silico* data set demonstrates how to accurately describe long-range distances within intrinsically disordered proteins, we aimed to test the validity of this approach on experimental data. For this, we used a 110 residue long protein from the disordered N-terminus of the measles virus phosphoprotein (P_1–100_). This protein has been extensively characterized by NMR spectroscopy^1,40^ and harbors two transient α-helices, as can be inferred from backbone chemical shifts (Figure 5D). We acquired nine FRET efficiencies, PREs from five different labeling sites (Figure 5A–C), a full set of backbone chemical shifts^1^ sensitive to local structural propensities, and SAXS reporting on the distribution of *R*_G_, i*.*e., the overall dimension of the protein. FRET efficiencies, obtained from random labeling of two engineered cysteines with Alexa488 and Alexa594 using maleimide chemistry, were recorded on a custom-built single-molecule fluorescence spectrometer. The corrected (see Methods for details) FRET histograms were fit with double-Gaussians describing populations at *E*_FRET_ = 0 (donor only population) and at *E*_FRET_ > 0, which was extracted for ensemble selection or cross-validation (Figure 5A, SI Figure 8 and SI Table 1). Comparison of the experimentally obtained FRET efficiencies with efficiencies expected from a flexible-meccano statistical coil ensemble suggests that P_1–100_ samples a conformational ensemble that is slightly more compact than a random coil.

Conformational ensembles comprising 200 conformers (see SI Figure 9 for an assessment of ensemble sizes) were selected using ASTEROIDS based on all PREs, chemical shifts (N, H_N_, C_O_, C_α_, C_β_), and six of the nine experimental *E*_FRET_. FRET efficiencies were included in the ensemble selection as described above, and the selected ensemble reliably reproduced the data used in the selection (Figure 5D, E, and F) as well as the four FRET efficiencies that have not been used in the selection (Figure 5F). A SAXS curve that was acquired from P_1–100_ and not used in the selection was also well described by the ASTEROIDS ensemble selected based on PREs, chemical shifts, and FRET efficiencies, suggesting that the ensemble also captured the overall dimension of the protein (Figure 5G). Analysis of the experimental SAXS curve as well as the SAXS curve back-calculated from the ensemble using extended Guinier analysis^41^ yielded comparable *R*_G_ values, which were also in agreement with the average *R*_G_ calculated directly from the selected conformational ensemble (SI Figure 10). The scaling exponent calculated from the selected ensembles, indicative of solvent quality, was determined to be 0.52, in agreement with θ-solvent conditions (SI Figure 10A) under which excluded volume interactions cancel out.^25,42^

Our experimental data combined with ASTEROIDS selections based on only FRET or only PREs show that long-range and intermediate- range distances of the conformational ensemble are only correctly sampled when combining both sets of data (SI Figure 11). This is in agreement with the theoretical complementarity of FRET and PREs regarding their sensitive distance ranges (Figure 3B), as shown on the example of an *in silico* data set (SI Figure 3). It is interesting to note that integration of PREs into the selection also improves the reproduction of two of the experimental FRET efficiencies, indicating that the FRET efficiencies alone might not sufficiently cover all relevant protein regions in the case of P_1–100_.

As, for this experimental data set, it is *a priori* not known on what time scale the fluorescent dyes sample the accessible volume, we additionally considered the other extreme case of AV sampling significantly faster than the fluorescence lifetime. FRET efficiencies of all conformers in the pool from which ensembles were selected were thus calculated under this assumption, and an ASTEROIDS selection was performed on the basis of six FRET efficiencies, five sets of PREs, and chemical shifts. This ensemble reproduces the FRET efficiencies not used in the selection less well than when slow (slower than the fluorescence lifetime) AV sampling was assumed (compare SI Figure 5B to Figure 5F). We thus conclude that “slow” AV sampling is appropriate for the P_1–100_ experimental FRET data. We note, however, that more rapid diffusion of fluorescent dyes has been observed for other experimental systems.^43,44^

As an additional cross-validation of both AV sampling and calibrations employed for the experimental smFRET experiments, we labeled one sample of P_1–100_ (C28–C64) with a different dye pair (Alexa488/Alexa647) and determined its FRET efficiency (SI Figure 12). In parallel, we simulated the Alexa488/Alexa647 dye pair onto the ensemble selected based on smFRET (Alexa488/Alexa594), PREs, and chemical shifts. The difference between experimental *E*_FRET_ (0.52) and *E*_FRET_ expected from the selected ensemble (0.56) is below the common error determined by a recent multilaboratory study.^45^

While, in all ASTEROIDS selections, an error of 0.02 for *E*_FRET_ was allowed in agreement with the measurement error over several independent measurements, a larger allowed error might be considered appropriate^45^ as the measured quantum yields, Förster distance *R*_0_, or determination of spectral crosstalk is also error prone. ASTEROIDS selections based on six FRET efficiencies, five sets of PREs, and chemical shifts allowing an error of 0.06, however, are in very good agreement with those selected allowing an error of 0.02 in the case of P_1–100_ (SI Figure 13).

### Reproduction of Experimental Fluorescence Lifetimes by Conformational Ensembles

In addition to intensity-based FRET efficiencies, calculated as a function of the number of emitted photons (cross-talk and background corrected; see Methods) of the donor (*I*_D_) and the acceptor (*I*_A_),
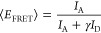
3and corrected for differences in quantum yield and detection efficiency in the green and red channel (γ), fluorescence lifetimes provide a complementary measure for distance distributions of a conformational ensemble.^46,47^ While, for a static donor–acceptor distance, *E*_FRET_ can be calculated from fluorescence lifetimes of the donor in the absence (τ_D_) and presence of the acceptor (τ_D(FRET)_; see also eq 2), this is not the case for distances with dynamics longer than the fluorescence lifetime and shorter than the interphoton time (usually on the order of tens of microseconds):^47,48^
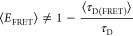
4Indeed, taking into account fluorescence lifetimes in the conformational ensemble of an IDP is complex, as every conformer in the ensemble contributes a single-exponential decay to the time-resolved fluorescence intensity of a time-correlated single photon counting (TCSPC) experiment, and the resulting multiexponential intensity decay is then experimentally convolved with the instrument response function (IRF) of the smFRET setup.^17^

In order to test whether the distance distributions of our conformational selection are in agreement with our experimental fluorescence lifetimes, we first extracted the fluorescence intensity decays of the FRET population from our single-molecule data (SI Figure 14A). The IRF was measured independently under the same experimental conditions, described with a double Gaussian function, and convoluted with the multiexponential decays expected for our conformational ensemble. The resulting decay curves described the experimental intensity decays remarkably well (Figure 5H, SI Figure 14B), indicating that our conformational ensemble correctly reproduces another set of independent long-range data that was not used in the ASTEROIDS selection process, thus confirming the validity of the selected ensemble as well as the time scales applied for motional sampling of both dyes and proteins within the ensemble.

## Discussion

A molecular description of the conformational landscape sampled by IDPs and proteins containing intrinsically disordered regions (IDRs) is of paramount interest, as IDPs and IDRs are enriched in several essential biological processes, such as signaling,^49,50^ cellular transport processes,^51,52^ and gene regulation,^53,54^ and their misregulation is often also linked to disease.^55^ Although multiconformational models have been conceived using mainly NMR and small angle scattering data,^29,39,56−58^ and in some individual cases single-molecule FRET efficiencies,^19,26^ those approaches fall short in integrating specific long-range and short-range information in a predictive manner.

We now demonstrate a tool-set to integrate the three most powerful techniques for the analysis of IDPs: NMR, SAXS, and single-molecule FRET. We show the integration of several FRET efficiencies into ensemble selections, and we reproduce them with confidence. We perform the selection using the experimentally obtained FRET efficiencies rather than their inferred distances and reproduce the corresponding fluorescence lifetimes.

Modeling of the fluorophores in terms of accessible volumes^12^ on top of the pool of conformers from which the ensembles are selected is key to allowing an integration of parameters from techniques that have different experimental requirements: the attachment of fluorophores or spin radicals for single-molecule FRET and PREs, or no labeling/stable isotope labeling for SAXS/NMR. This approach assumes that the conformational ensemble remains quasi-identical in the presence and absence of the different labels (FRET/PRE) and that the parametrization of the AVs accurately reproduces the volumes sampled by the fluorophores. Successful cross-validation of a number of FRET efficiencies (including one with a different dye pair) not used in the selection and a SAXS curve suggest that these assumptions are indeed correct. The selection of explicit ensembles combined with the *in silico* attachment of labels also allows for its use if complex distance distributions are sampled that include transiently folded protein regions or even entire folded domains.^1,49,59^ Distance distributions within the protein backbone can be directly calculated from the selected ensemble. While we employ the genetic algorithm ASTEROIDS^27^ to select conformational ensembles in agreement with the experimental data, our developments concerning the integration of fluorophore AVs into conformational ensembles as well as insights into sampling of (sufficient) FRET distances along the protein sequence can also be used with other ensemble selection approaches.^19,23,26^

Importantly, we show that we can reproduce not only the FRET efficiencies that were used for the ensemble selection and cross-validate additional FRET efficiencies but also their corresponding fluorescence lifetimes. As fluorescence lifetimes of a FRET sample also depend on the distance distribution between the two attached fluorophores^47^ and have thus frequently been used in the analysis of folded as well as intrinsically disordered proteins,^17,33,48,60−62^ these results are particularly remarkable testifying to the predictive nature of our ensembles by reproducing an independent data set.

We show that PREs and FRET efficiencies provide complementary intermediate- and long-range information on the conformational ensemble, and it is worth noting that the ensembles selected on the basis of chemical shifts, PREs, and FRET efficiencies also reproduce an independently measured SAXS curve. This shows that these fundamentally different experimental techniques effectively agree with each other, therefore also supporting recent advances resolving^19,23^ the long-lasting controversy concerning compaction of IDPs measured by smFRET and SAXS.^19−24^

Apart from contributing distance ranges much longer than those accessible by PREs, including smFRET into the calculation of conformational ensembles of IDPs or proteins comprising intrinsically disordered regions has far-reaching consequences regarding the applicability of ensemble calculation: Since smFRET is not limited by the size of the protein, nor any dynamic time scale sampled by the protein, FRET efficiencies can also be measured under conditions where NMR line broadening leads to factual disappearance of the signal.^33,63^ Furthermore, the low protein concentrations used in an smFRET experiment (in the picomolar range) also allow accessing aggregation-prone proteins^54,64^ or performing experiments within the cell under physiological conditions.^10,11^ Using FRET efficiencies for the calculation of conformational ensembles thus allows addressing the conformational landscape of IDPs under conditions that are not accessible by any other technique.

## Conclusion

With the integrated use of NMR, SAXS, and single-molecule FRET to calculate multiconformational models that satisfy all data, we now demonstrate how different experimental techniques can synergize to reliably describe IDPs, and we demonstrated this on the example of the measles virus phosphoprotein. With the increasing awareness of the importance of IDPs, in particular also in liquid–liquid phase separation,^65,66^ we expect this tool-set to make an important impact in integrative multiconformational modeling of dynamic systems.

## Materials and Methods

### Accessible Volume Calculations

AV calculations were based on procedures described previously.^12,36^ Briefly, positions that the dyes are expected to sample were calculated considering a linker length, as well as three radii (*R*_1_, *R*_2_, *R*_3_). Pairwise distances between the positions sampled by the donor and the acceptor fluorophore were then calculated with a coarsening step size of 200 with respect to the position list. Distance histograms as well as average FRET efficiencies were compared over an ensemble of 200 conformers using a step size of 10, 50, and 200.

For the calculation of FRET efficiencies on large conformational ensembles, the calculation speed of the AV had to be optimized: Positions describing the accessible volumes were sampled in an iterative way. A total of 100, 500, 1000, and 10 000 iteration steps were tested for reproducibility of distance histograms and average FRET efficiencies over a 100 conformer ensemble. A total of 500 iterations led to sufficiently accurate distance histograms that reproduce FRET efficiencies reliably.

In order to avoid “mutating” amino acids into cysteines *in silico*, AVs were calculated from the CB atom of the respective amino acid. The linker length in the simulations was optimized to take the distance between CB and SH of a cysteine into account. The estimate (*L* = 22.83 Å) was based on geometrical considerations, and an ensemble by which the AV was calculated from the CB as attachment point has been verified to reproduce the distance histograms and FRET efficiencies calculated from the same ensemble, but with SH as attachment point (*L* = 21 Å).

**Table 1:** 

	Alexa488	Alexa594	Alexa647
*L*	22.83	22.83	22.83
*R*_1_	6.8	7.6	11
*R*_2_	3.9	4.1	4.7
*R*_3_	1.5	2.2	1.5

Scripts provided by Walczewska-Szewc et al.^36^ have been adapted to contain the changes above. Attachment points were read from the respective PDB files of the conformational ensemble in an automated way using in-house software and were then used for the calculation of AVs and distance histograms. Parametrization for Alexa647 was adapted from Peter et al.^67^ PDB files containing full side chains were used for AV calculation. Conformer-wise FRET files were then generated as an input for ASTEROIDS^27,28^ selection, containing the different FRET distances used in the selection.

### Incorporation of FRET Efficiencies into Multiconformational Models

AVs were calculated per conformer as described. Pairwise distances between the sampled volumes of the two fluorophores are calculated and converted into FRET efficiencies according to
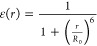
5with the Förster distance *R*_0_ and the distance *r* between the sampled points in the AV. The average FRET efficiency of one conformer is then calculated as the average of ε over all pairwise distances *n*:
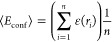
6in accordance with a sampling of the AV on a time scale significantly longer than the fluorescence lifetime. The average FRET efficiency of the ensemble comprising all conformers (which is then compared to the measured *E*_FRET_) can be described as
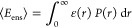
7with ε(*r*) as described in eq 5 and *P*(*r*) describing the distance distribution containing all pairwise distances of the AVs for every conformer. Computationally, for an ensemble of *m* conformers, ⟨*E*_ens_⟩ can be calculated as
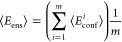
8with ⟨*E*_conf_^*i*^⟩ describing the average FRET efficiency of the *i*th member of the ensemble as described in eq 6.

For considerations assuming a sampling of the AV that is much faster than the fluorescence lifetime, pairwise positions of the fluorophores were first determined and their sixth power was calculated and then averaged per conformer.^68,69^ The FRET efficiency was calculated from these averaged distances on a conformer-by-conformer basis:
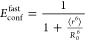
9*R*_0_ used in the calculations was determined experimentally. The quantum yield of P_1–100_ labeled with Alexa488 was determined by the comparative method^70^ described previously with fluorescein (in 0.1 M NaOH, Φ = 0.95, *n* = 1.334)^71^ and Rhodamine 6G (in ethanol, Φ = 0.94, *n* = 1.361)^72^ as quantum yield standards. The overlap integral *J*(λ) was determined from P_1–100_ samples labeled with Alexa488 and Alexa594.^46^ Rapid orientation averaging of the fluorescent dyes was assumed, leading to the common assumption of κ^2^ = ^2^/_3_. Fluorescence anisotropies measured on the different P_1–100_ samples suggested that this assumption was valid (SI Table 2). *R*_0_ was then calculated according to^46^
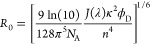
10with the Avogadro number *N*_A_, the overlap integral *J*(λ), the orientation factor κ^2^, the quantum yield of the donor in the absence of acceptor Φ_D_, and the refractive index *n*. *n* = 1.3 was used for P_1–100_ in its measurement buffer. An *R*_0_ of 56 Å was obtained for the dye pair Alexa488/Alexa594 in 50 mM Na-phosphate pH 6, 150 mM NaCl, and 2 mM dithiothreitol (DTT). The same *R*_0_ was used to compute the *in silico* data set.

### Generation of *in Silico* Ensemble

Flexible-meccano^29^ was used to generate a large conformational ensemble (10 000 conformers) of a 155 amino acid long IDP. The centers of mass of all C_α_ atoms from residues 15–25 as well as residues 90–100 were calculated, and all conformers with a distance of less than 20 Å between these centers of mass were selected. AVs of Alexa488 and Alexa594 were computed as described above for 15 *in silico* “labeling positions” (SI Figure 2), and FRET efficiencies for this ensemble comprising a long-range contact were calculated as described above and used as an input for ASTEROIDS or for cross-validation of ensembles selected using ASTEROIDS. Expected PREs for this ensemble were calculated as described elsewhere (labeling sites were residues 23, 65, 70, 92, and 130).^39^ τ_c_ and τ_e_ were set to 5 and 0.5 ns, respectively. τ_C_ = τ_r_τ_s_/(τ_r_ + τ_s_) describes the rotational correlation time of the protein (τ_r_) and the effective electron relaxation time (τ_s_), and τ_e_ = 1/(τ_i_^–1^ + τ_r_^–1^ + τ_s_^–1^) depends on the effective correlation time of the spin label (τ_i_) according to a model-free expression of the spectral density function.^38,39^^1^H *R*_2_ relaxation was assumed to be 18 s^–1^ throughout the protein.

### Selection of Conformational Ensembles

Ensembles of 200 conformers were selected from a large statistical coil ensemble (10 000 conformers), generated through flexible-meccano,^29^ using the genetic algorithm ASTEROIDS.^27^ Selection based on PREs and chemical shifts was performed as described previously.^39^ Selection based on FRET efficiencies allowed an error of 0.02 and was weighed 50% as compared to an NMR experiment (e.g., all PREs arising from one spin labeling site).

For P_1–100_, ensembles were first selected based on only chemical shifts during four iterations of flexible-meccano/ASTEROIDS. A large conformational ensemble (10 000 conformers) was then calculated based on the resulting Φ/Ψ angles, from which subensembles were selected based on FRET, PREs, and chemical shifts. FRET efficiencies not used in the selection were back-calculated as described above. SAXS curves were back-calculated using CRYSOL.^73^ Chemical shifts were calculated using SPARTA.^74^

### Back-Calculation of Fluorescence Lifetimes

Distance distributions between the donor and acceptor fluorophores were calculated from the selected conformational ensembles, and the corresponding fluorescence lifetime decays were calculated as^23^
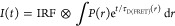
11with the instrument response function (IRF) experimentally determined and described by a double Gaussian function, and the fluorescence lifetime of the donor in the presence of the acceptor τ_D(FRET)_ calculated for every distance *r* according to
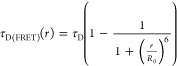
12

The fluorescence lifetime of the donor in the absence of the acceptor (τ_D_) and the Förster distance (*R*_0_) were experimentally determined. A scattering contribution was added to the fluorescence lifetime decays, and both decay and scattering were scaled independently to best fit the experimental data.

### Protein Production

P_1–100_ tagged with 8 His was expressed and purified as described earlier.^1,40^ Briefly, a pET41c(+) plasmid containing P_1–100_ was transformed into Rosetta (λDE3)/pRARE (Novagen), and cultures were grown at 37 °C in lysogeny broth (LB) medium until an optical density (OD) of >0.6. Expression was induced with 1 mM isopropyl-β-d-thiogalactopyranoside and continued at 20 °C overnight. Cells were lysed by sonication in 20 mM Tris pH 8/150 mM NaCl and purified using standard Ni purification. The protein was eluted from the Ni resin by adding 400 mM imidazole to the lysis buffer. The protein was then further purified on a Superdex 75 column (GE Healthcare) in 50 mM Na-phosphate, pH 6, 150 mM NaCl, and 2 mM DTT. Expression of protein labeled with ^15^N followed the same procedure, except that the protein was expressed in M9 minimal medium. All experiments were conducted in 50 mM Na-phosphate pH 6, 150 mM NaCl, and 2 mM DTT. DTT was not contained in buffers used for PRE experiments.

### Protein Labeling with Fluorophores or Spin Radical Labels

P_1–100_ was randomly labeled with Alexa488 and Alexa594 essentially as described previously.^8,75^ Briefly, 20 mM DTT was added to the protein sample and incubated overnight at 4 °C. The protein was then dialyzed into degassed 50 mM Na-phosphate pH 7 and 150 mM NaCl buffer until all DTT was washed out. Alexa488 and Alexa594 were added simultaneously at an excess of approximately 5× compared to protein. Labeling was allowed to proceed 30 min at room temperature, followed by 4 °C overnight. The labeled protein was then separated from excess dye by size exclusion chromatography on an Enrich SEC70 (Biorad) column using 50 mM Na-phosphate buffer (pH 6), 150 mM NaCl, and 2 mM DTT.

Labeling of ^15^N P_1–100_ single cysteine mutants for PREs was achieved using *S*-(1-oxyl-2,2,5,5-tetramethyl-2,5-dihydro-1*H*-pyrrol-3-yl)methylmethanesulfonothioate (MTSL) and followed essentially the same procedure as for fluorescence labeling. The final buffer used for size exclusion chromatography, however, did not contain DTT.

### Experimental NMR Data

All NMR experiments were performed at a temperature of 19 °C. The assignment of P_1–100_ was obtained previously^1^ and used as an input for ASTEROIDS^39^ selection as well as for calculation of secondary chemical shifts and secondary structure propensities^76^ (SSPs).

For calculation of PREs, HSQC spectra of the different Cys mutants of ^15^N P_1–100_ were measured in the presence and absence of the MTSL label. Spectra were processed with NMRPipe,^77^ peak intensities were extracted from the respective spectra, and the ratio between MTSL labeled and unlabeled peak intensities was determined and used as an input for ASTEROIDS.

### Experimental Single-Molecule FRET data

Single-molecule fluorescence spectroscopy was measured on a custom setup built around an Olympus IX73 microscope equipped with a 60× water immersion objective (NA 1.2). A pulsed laser diode (40 MHz, LDH 485, Picoquant, Berlin, Germany) was fed through a λ/4 plate and focused onto the sample to excite freely diffusing P_1–100_ molecules with circularly polarized light. Fluorescence emission was spatially filtered through a pinhole with a 100 μm diameter, separated into green (Alexa488) and orange (Alexa594) fluorescence, and focused onto two PMA hybrid detectors (Picoquant). Photons were counted using a Hydraharp (Picoquant). smFRET experiments were performed at room temperature.

FRET histograms were calculated using custom code written in Python. Lists of photon arrival times were first extracted using a code written in C, adapted from a demo-code provided by Picoquant.^78^ Photon streams were then binned with a 1 ms bin width and subjected to a Lee filter before bust integration and thresholding.^79^ A threshold of at least 50 photons was used. Fluorescence intensities were corrected for background contribution, spectral crosstalk, differences in quantum yield (determined as described previously^70^), and differences in detection efficiencies between the green and the orange channel.

Microtimes were extracted for bursts corresponding to the FRET peak and to the 0-FRET peak separately, and population averaged lifetime histograms were built. The instrument response function was measured on buffer under the same conditions as the single-molecule experiments, and lifetimes of the donor were extracted through fitting the lifetime histograms of the 0-FRET population with a single-exponential function convolved with the IRF.

### Corrections Employed in the smFRET Experiments

Buffer background was measured using the same conditions as for the single molecule experiments, and bin-wise background contributions were determined for the donor and acceptor channel and subtracted from the bin-wise photon counts in the single-molecule FRET experiments.

Differences in detection efficiencies and quantum yields were included in the correction factor γ (see eq 3:
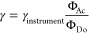
13with  being the difference in detection efficiency between acceptor (η_Ac_) and donor (η_Do_) signal of the instrument determined as described in Ferreon et al., 2009.^80^ Briefly, fluorescence of free donor and acceptor dyes in the measurement buffer was measured on an ensemble fluorescence spectrometer (PTI Quantamaster) and on the single-molecule fluorescence setup at the same excitation wavelength. Ensemble fluorescence spectra were corrected for detection differences at different wavelengths, and the total signal was extrapolated to the full emission spectra. Plots displaying the integrated ensemble fluorescence versus fluorescence recorded on the single-molecule setup were fitted with a line for donor and acceptor fluorescence independently. The ratio of the slopes (*m*_Ac_/*m*_Do_) was determined to be γ_instrument_ and is 0.81 for the Alexa488/Alexa594 dye pair and 0.83 for the Alexa488/Alexa647 dye pair in 50 mM Na-phosphate pH 6, 150 mM NaCl, and 2 mM DTT. The spectral properties of fluorescently labeled P_1–100_ were equal to those of the free dyes in the same buffer. γ_instrument_ was corrected on a daily basis based on a short measurement of Rhodamine 6G,^46^ which emits into the donor and acceptor channel of the smFRET setup.

Fluorescence quantum yields of the donor (Φ_Do_) and acceptor (Φ_Ac_) were determined from singly labeled P_1–100_ proteins in the measurement buffer using the comparative method described by Williams et al. as described above.^70^ Rhodamine 101 (in ethanol, Φ = 1.0, *n* = 1.36)^81^ was used as a quantum yield standard for Alexa594-labeled proteins (see section Incorporation of FRET Efficiencies into Multiconformational Models for standards used for Alexa488-labeled proteins). For Alexa647-labeled proteins (SI Figure 12), cresyl violet (in ethanol rather than methanol, Φ = 0.54, *n* = 1.33)^82^ was added as an additional quantum yield standard. A refractive index of *n* = 1.3 was used for all P_1–100_ samples. The quantum yields determined for the different P_1–100_ single cysteine constructs were very similar, and their average quantum yields were thus used both for γ correction and for the calculation of the Förster distance (*R*_0_).

Leakage was determined from measurements undertaken in the context of γ correction by calculating the ratio of donor fluorescence arriving in the acceptor versus the donor channel of smFRET setup. These values were corrected on a daily basis using the Rhodamine 6G calibration measurement and validated by ensuring that the donor-only peak of the single-molecule FRET histograms was situated at a FRET efficiency of 0.

To estimate the contribution of direct excitation, an IDP sample labeled with Alexa488 and Alexa594 separated by 164 amino acids was prepared, which is not expected to yield *E*_FRET_ > 0.^46^ While we cannot entirely exclude that this is indeed not the case, the contribution of direct excitation was tentatively attributed to be 0.2 photon per 1 ms under this assumption. Since application of this correction yields differences in *E*_FRET_ of only around 0.01 to 0.03, we decided not to apply this correction. This remains true if the ratio of extinction coefficients between the donor and acceptor at the excitation wavelength is used to correct for direct excitation.^83,84^ In order to test the validity of this approximation, the DNA sample “4-mid”, labeled with Atto488/Atto594 used in Hellenkamp et al., 2018,^45^ has been measured and corrected using the same procedure (SI Figure 12B, γ was determined independently, and quantum yields as well as *R*_0_ were used as described by atto-tec^85^), leading to *E*_FRET_ = 0.39 compared to 0.41 ± 0.04 as reported by Hellenkamp et al.^45^

### Experimental SAXS Data

SAXS experiments were measured for five different concentrations of P_1–100_ from 0.5 to 2 mg/mL at 20 °C on BM29 at the European Synchrotron Radiation Facility (ESRF), Grenoble, France. Scattering was measured at a wavelength of 0.992 Å, and samples were exposed during 10 frames. Frames not impacted by radiation damage were averaged. Buffer scattering curves were subtracted from the scattering curves of P_1–100_.

### Theoretical Comparison between FRET and PRE Rates

Figure 3A–C were generated considering a static measured distance for both FRET (*k*_ET_) and PRE rates (*R*_2,PRE_).
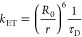
14was calculated with a Förster distance (*R*_0_) of 56 Å and a fluorescence lifetime of the donor (τ_D_) of 4 ns. *E*_FRET_ was calculated from *k*_ET_ as described in eq 2. *r* is the distance between donor and acceptor fluorophores.^46^

The PRE transverse relaxation rate (*R*_2,PRE_) was calculated according to
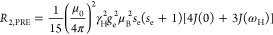
15with the electron *g*-factor *g*_e_, the gyromagnetic ratio of the observed proton γ_H_, the electron spin *s*_e_, the Bohr magneton μ_B_, and the proton frequency ω_H_.

A spectral density function of
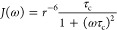
16was used with τ_c_ = τ_r_τ_s_/(τ_r_ + τ_s_), τ_r_ being the rotational correlation time of the protein and τ_s_ the effective electron relaxation time. τ_c_ was set to 5 ns for Figure 3C. *r* is the distance between the ^1^H_N_ nuclei and the PRE label.^38,39^

Note that for the calculation of PREs in the context of a conformational ensemble of an IDP, a model-free expression of the spectral density function was used, describing the internal motion of the IDP as well as the motion of the spin label:
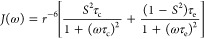
17

The order parameter *S*^2^ denotes the motion of the dipolar interaction vector, τ_c_ is as described above, and τ_e_ = 1/(τ_i_^–1^ + τ_r_^–1^ + τ_s_^–1^) additionally depends on the effective correlation time of the spin label (τ_i_).^39^

## Supporting Information Available

Additional figures showing a comparison between the *in silico* attachment points of accessible volumes, an overview of the protein constructs used for the *in silico* data set, ASTEROIDS selection using only *in silico* FRET or PREs, different numbers of FRET efficiencies, different numbers of conformers, and different allowed errors, comparison of different dye averaging regimes, FRET histograms of P_1–100_, description of fluorescence lifetimes by an ASTEROIDS ensemble of P_1–100_, validation samples (DNA and P_1–100_ labeled with a different dye pair), fits of P_1–100_ SAXS data, as well as two tables summarizing the experimental fluorescence data and FRET efficiencies obtained from selections (PDF)
