# Supplementary material for: Quantitative Description of Intrinsically Disordered Proteins Using Single-Molecule FRET, NMR, and SAXS
Source: J Am Chem Soc. 2021 Nov 24;143(48):20109–21. doi: 10.1021/jacs.1c06264 (PMC8662727; doi:10.1021/jacs.1c06264)
Supplement: Supplementary file 1 — ja1c06264_si_001.pdf [file ja1c06264_si_001.pdf]

Supporting Information

Quantitative description of intrinsically disordered proteins using single-molecule FRET, NMR and SAXS

Samuel Naudi-Fabra[1], Maud Tengo[1], Malene Ringkjøbing Jensen[1], Martin Blackledge[1], Sigrid Milles[1,*]

[1]Institut de Biologie Structurale, Université Grenoble Alpes-CEA-CNRS, 71, Avenue des Martyrs, 38044 Grenoble, France.

*correspondence to : sigrid.milles@ibs.fr

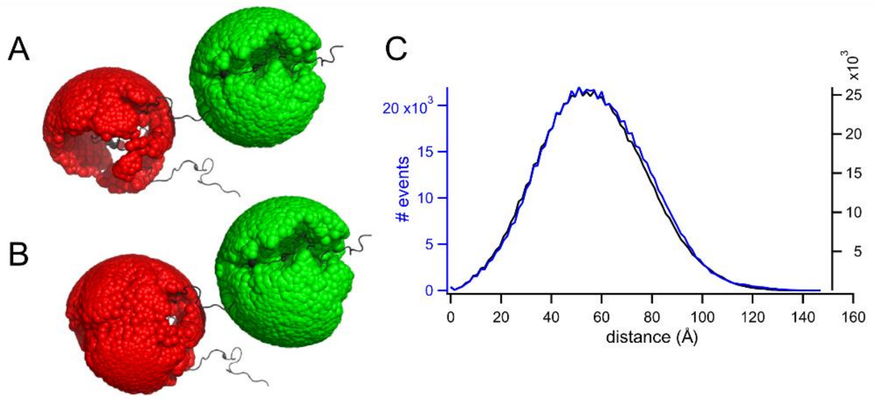

SI Figure 1: Attachment point of accessible volumes. (A) Accessible volumes calculated starting from the Cys S_H atom according to Walczewska-Szewc et al.[1] **(B)** Accessible volumes calculated starting from the C_β atom of the respective cysteines. **(C)** Comparison of distance histograms over a conformational ensemble of 100 conformers.

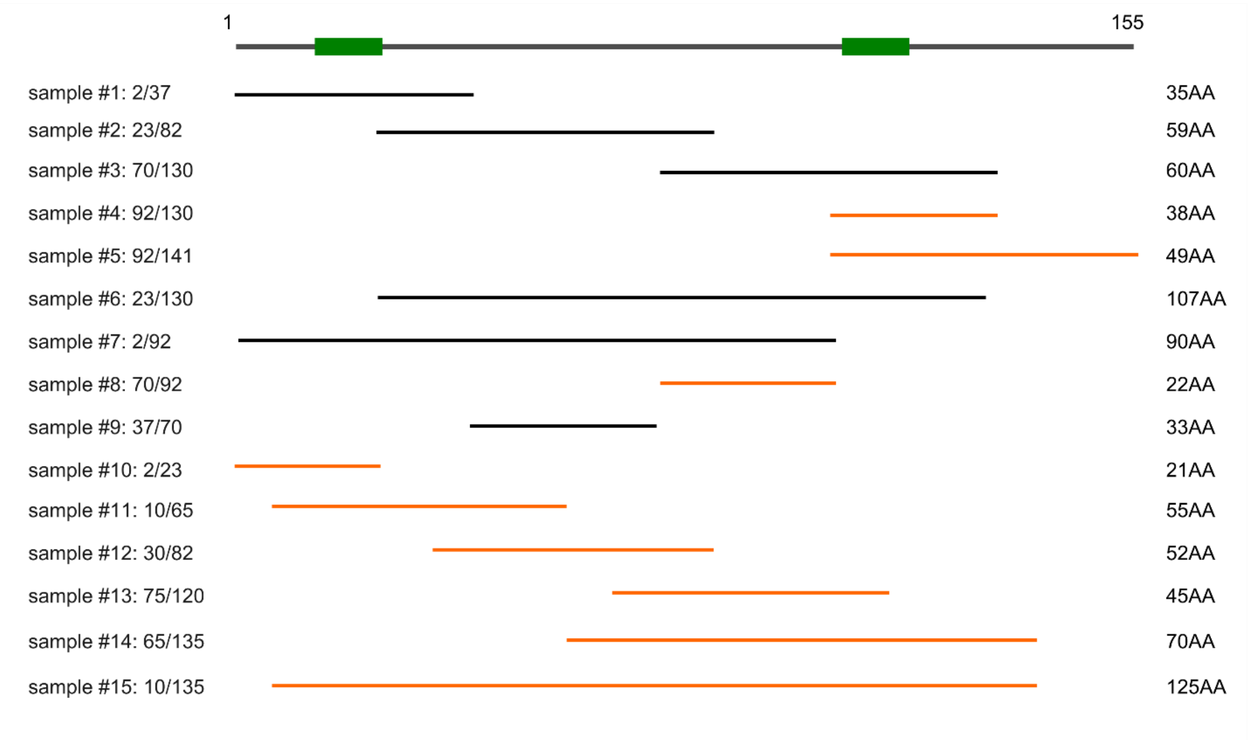

SI Figure 2: Constructs used for the *in silico* data. Top: Scheme of a 155 residue long IDP with a long range contact of less than 20 Å between amino acids 15-25 and 90-100. Below are shown the different samples for which FRET efficiencies have been calculated. Calculations have always been performed using the full length protein, the numbers (left) indicate the positions to which the fluorophores were attached. On the right, the amino acid distance between the attachment points is shown. Black constructs have been used in the selection. Orange constructs have been used for cross-validation.

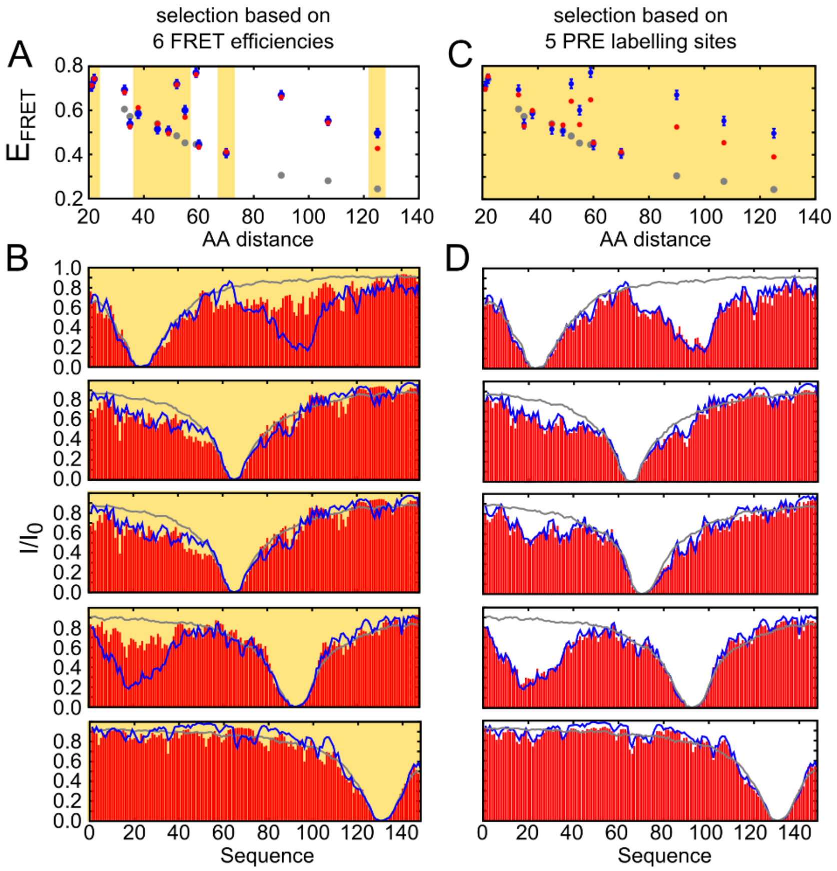

SI Figure 3: Complementarity between FRET and PREs. (A) ASTEROIDS[2,3] selection based on 6 FRET efficiencies (E_{FRET}) of the long-range *in silico* ensemble described in Figure 3. E_{FRET} are plotted against amino acid distance between the attached labels. Gray: Values expected for a statistical coil ensemble (flexible-meccano[4]); blue: values calculated from the *in silico* data. Error bars are 0.02 and depict the allowed error in the ASTEROIDS selection. Red: Values calculated from the selected ensemble. **(B)** PREs back-calculated from the selection based on 6 E_{FRET} (red), as expected from a flexible-meccano statistical coil (gray) and calculated from the *in silico* long-range ensemble (blue). Shown are intensity ratios between the reduced and the oxidized state of the spin label (I/I_0) **(C)** ASTEROIDS selection based on 5 different PRE labelling sites of the long-range *in silico* ensemble described in Figure 3. E_{FRET} were not used in the selection. Colour code as in (A). **(D)** PREs that were used in the selection. Colour code as in (B). Data above yellow background were not used in the selection.

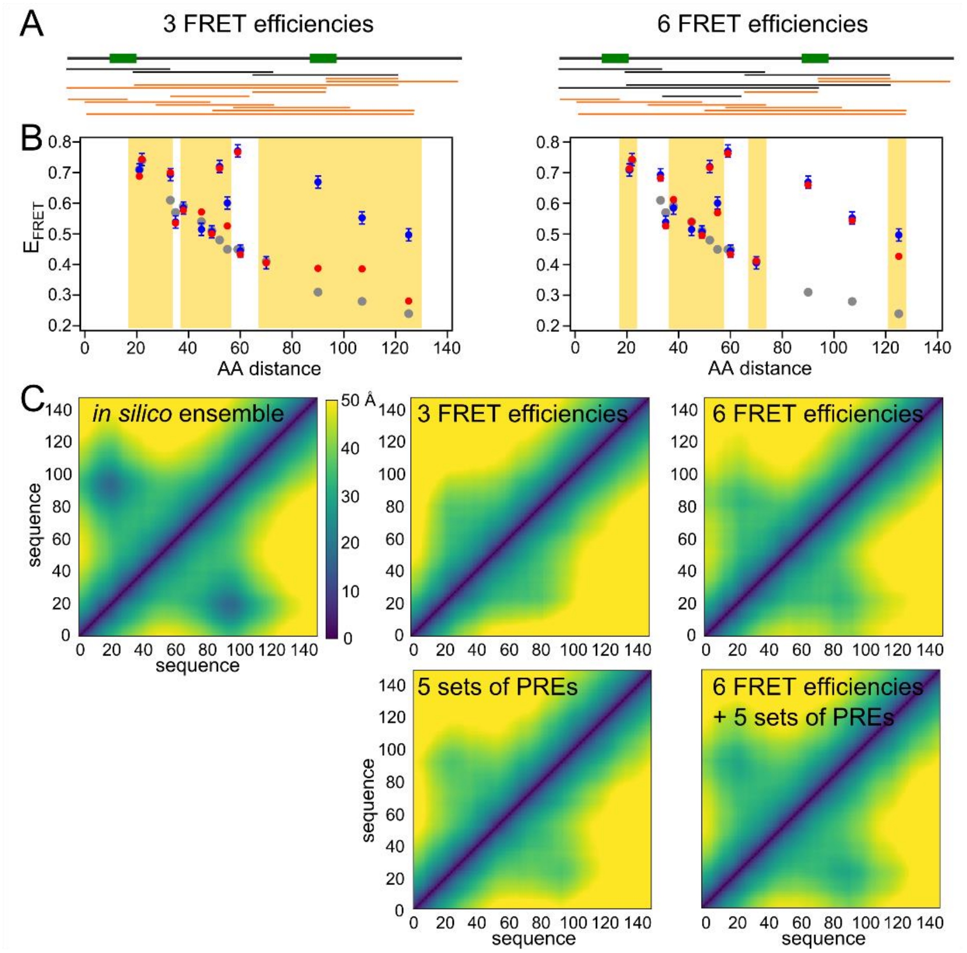

SI Figure 4: Effect of number and type of ensemble selection input on reproduction of the target. (A) Scheme of the model protein with a long-range contact (green boxes) and below the *in silico* FRET constructs. The constructs used for cross-validation are in orange. (B) FRET efficiencies (E_{FRET}) versus amino acid (AA) distance for the original flexible-meccano ensemble (grey points), the *in silico* target ensemble (blue) and errors allowed in the selection, and the selected ensemble (red) using 3 (left) and 6 (right) FRET efficiencies for the selection, respectively. (C) Contact maps of the *in silico* target ensemble (upper low left) and selections based on 3 FRET efficiencies (upper row, middle), 6 FRET efficiencies (upper row, right), 5 sets of PREs (lower row, middle), and 6 FRET efficiencies plus 5 sets of PREs (lower row, right).

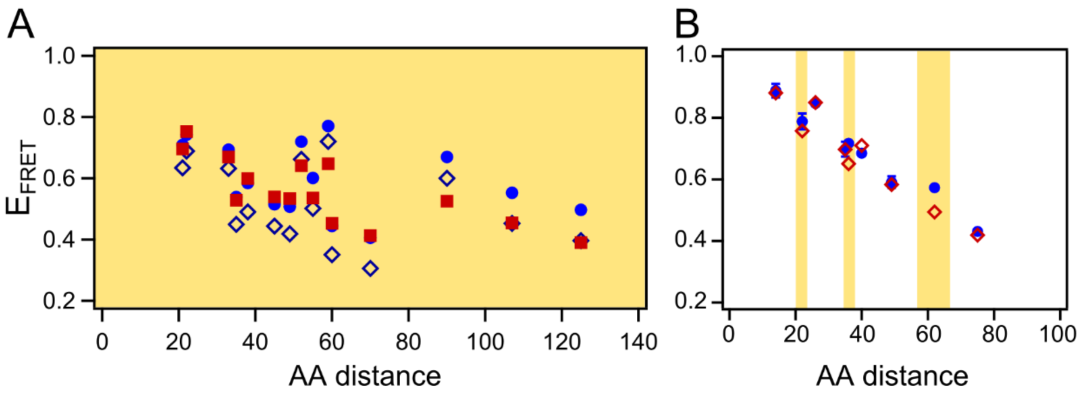

SI Figure 5: Comparison of dye averaging regimes. (A) E_{FRET} plotted against amino acid (AA) distance between the two labelling sites for the long-range *in silico* ensemble described in Figure 3 using an averaging regime of dye accessible volume sampling longer than the fluorescence lifetime (filled blue points) and shorter than the fluorescence lifetime (open, dark blue diamonds). Red, filled squares are FRET efficiencies back-calculated from an ensemble that has been selected based on only PREs under the assumption of AV sampling significantly faster than the fluorescence lifetime. (B) E_{FRET} plotted against amino acid (AA) distance between the two labelling sites for the experimental FRET efficiencies of P_{1-100} (blue points, error bars are standard deviations of repeated measurements) and for FRET efficiencies calculated from an ASTEROIDS ensemble selected based on 6 FRET efficiencies and 5 sets of PREs (red open diamonds). FRET efficiencies of the pool from which conformers are selected as well as those back-calculated from the selected ensemble were determined under the assumption of AV sampling significantly faster than the fluorescence lifetime. Data not used in the selection are plotted above yellow background (A and B).

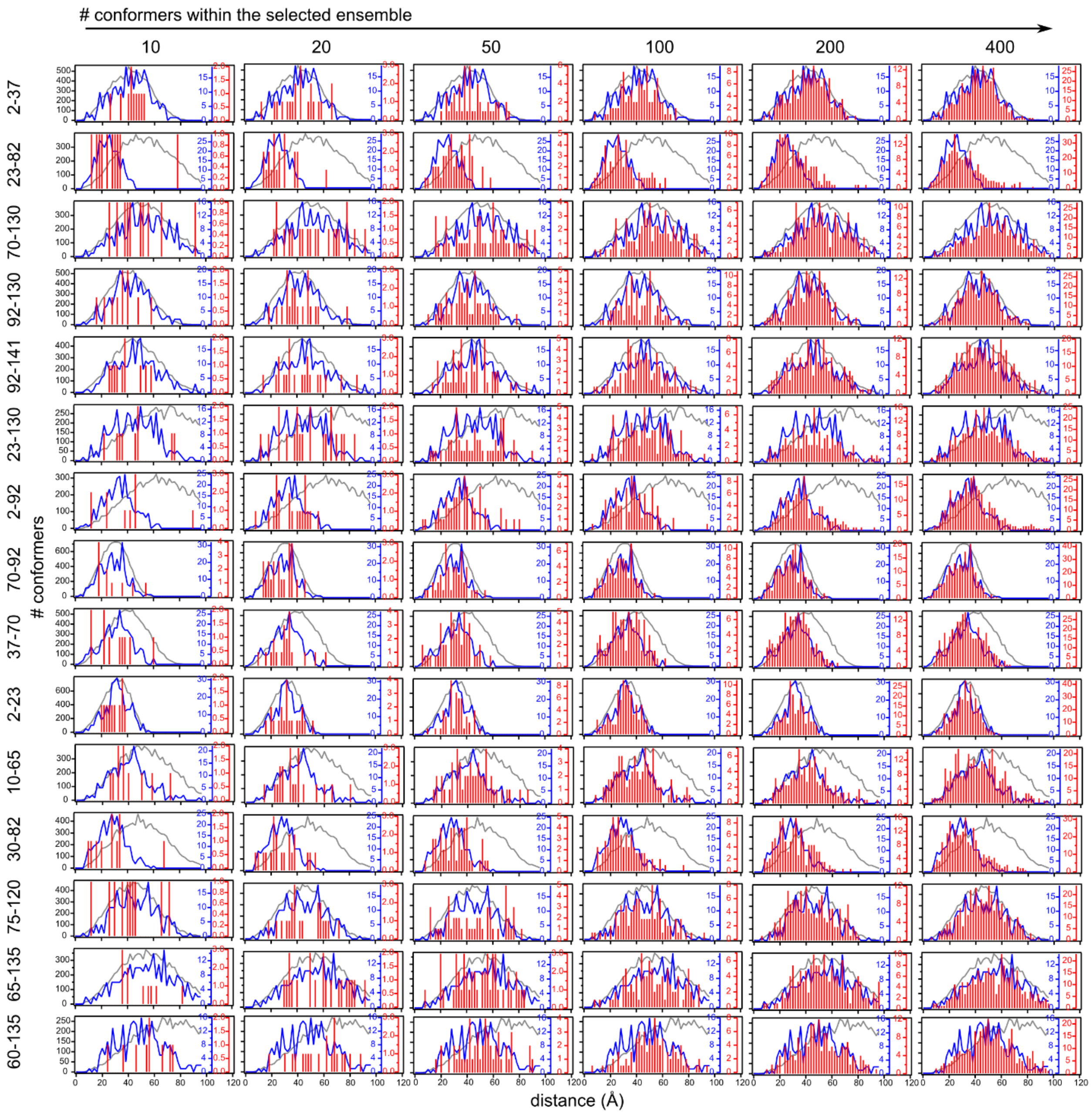

SI Figure 6: C_α- C_α distances between the *in silico* labelling sites for different ensemble sizes. In red are the distances calculated from one selection based on 6 FRET efficiencies and using 5 PRE labelling sites. The expected C_α- C_α distances are shown in blue, the distances obtained from a flexible-meccano statistical coil ensemble in gray. Top to bottom: Different labelling sites; the numbers refer to the dye attachment sites (number of amino acid in the sequence). Left to right: increasing number of conformers in the selected ensemble: 10, 20, 50, 100, 200 and 400.

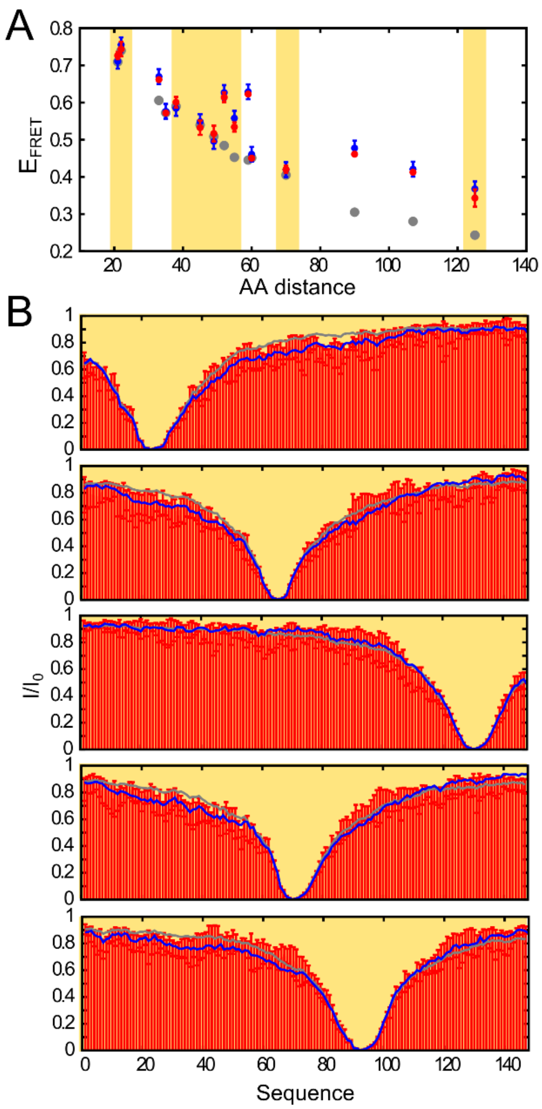

SI Figure 7: Ensemble selected based on *in silico* data with long range contact smaller than 50 Å. 6 independent ensembles were selected based on 6 FRET efficiencies (E_{FRET}). (A) E_{FRET} versus amino acid (AA) distance of the label attachment points. Gray: statistical coil ensemble (flexible-meccano); blue: *in silico* data, error bars denote the error allowed in the selection (0.02), red: average E_{FRET} from the 6 independent selections, error bars are corresponding standard deviations. (B) PREs back calculated from the selected ensembles (red, averages of 6 selections and error bars are corresponding standard deviations); Blue: PREs calculated from the in silico ensemble; Gray: PREs calculated from the selection pool (statistical coil, flexible-meccano).

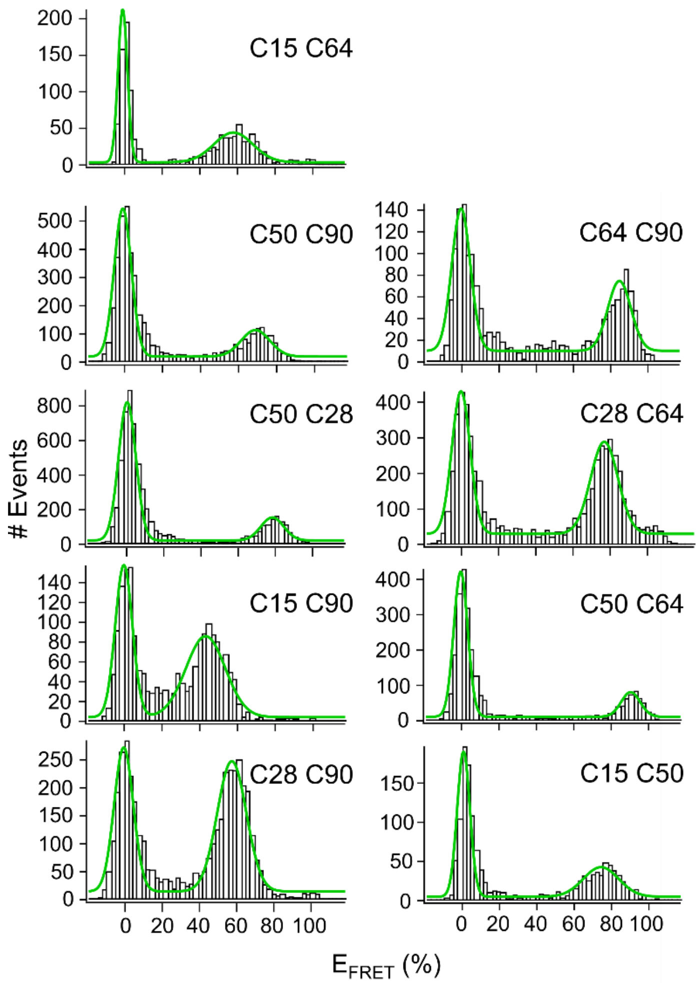

SI Figure 8: FRET histograms of P_{1-100}. FRET histograms were calculated from smFRET data of the different labelling constructs of P_{1-100} (black bars) and fit with a double Gaussian function (green) to extract the E_{FRET} of the non-zero FRET peak.

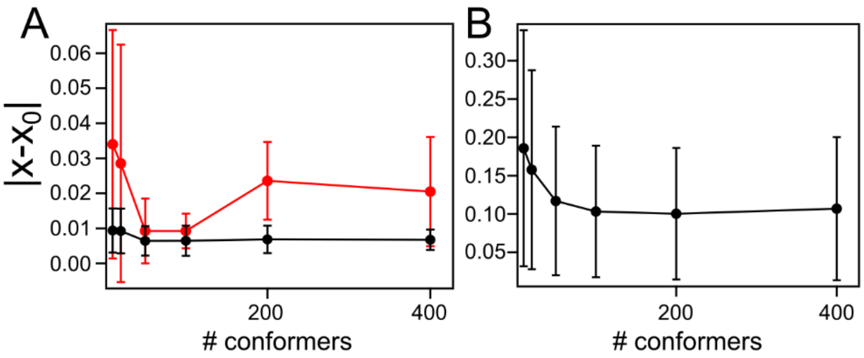

SI Figure 9: Analysis of ensemble sizes. Averaged absolute deviations (|x-x_0|) of FRET efficiencies (A) and PREs (B) between selected ensembles and experimental data of $P_{1\text{-}100}$. Ensemble sizes were 10, 20, 50, 100, 200 or 400 conformers. Three independent selections for every ensemble size were included in the average. Red points illustrate data not used in the selection. Lines connecting the points are shown. While 50 or 100 conformer ensembles seem to reproduce FRET efficiencies not used in the selection slightly better, a size of 200 conformer still reproduces those passive data very well and provides a better statistical description of the ensemble (see also SI Figure 6).

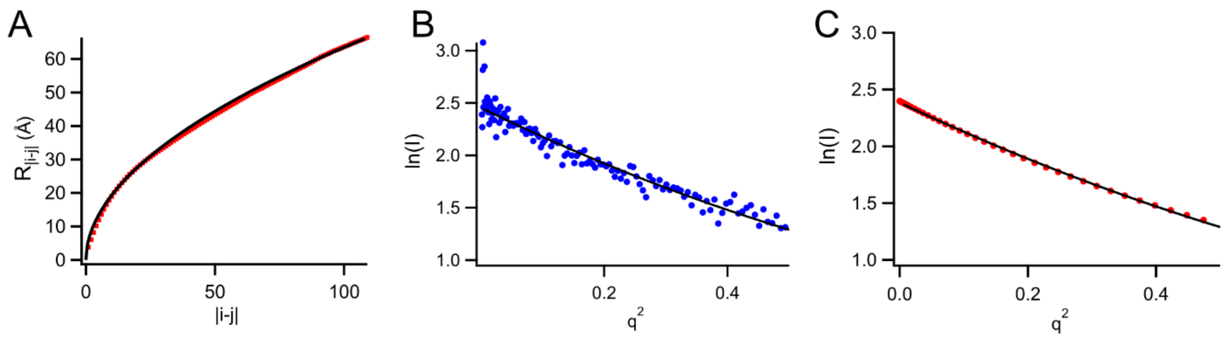

SI Figure 10: Radii of gyration determined from SAXS and the selected ensemble. (A) Pairwise C_α-C_α distances were calculated from an ASTEROIDS ensemble of $P_{1\text{-}100}$ calculated based on 6 FRET efficiencies, 5 sets of PREs and chemical shifts. The distances were plotted against the amino acid difference (red points) and fit to $R_{|i-j|}=a*|i-j|^{\nu}$ with a being a pre-factor and ν the scaling exponent (black curve). ν was determined to be 0.52 by the fit. (B) Experimental SAXS data (blue points) and extended Guinier fit (black curve) using the scaling exponent determined in (A), lead to an R_G of 2.9 nm. (C) SAXS curve calculated back from the ASTEROIDS ensemble (red points) and extended Guinier fit (black curve) using the scaling exponent determined in (A), lead to an R_G of 2.8 nm.

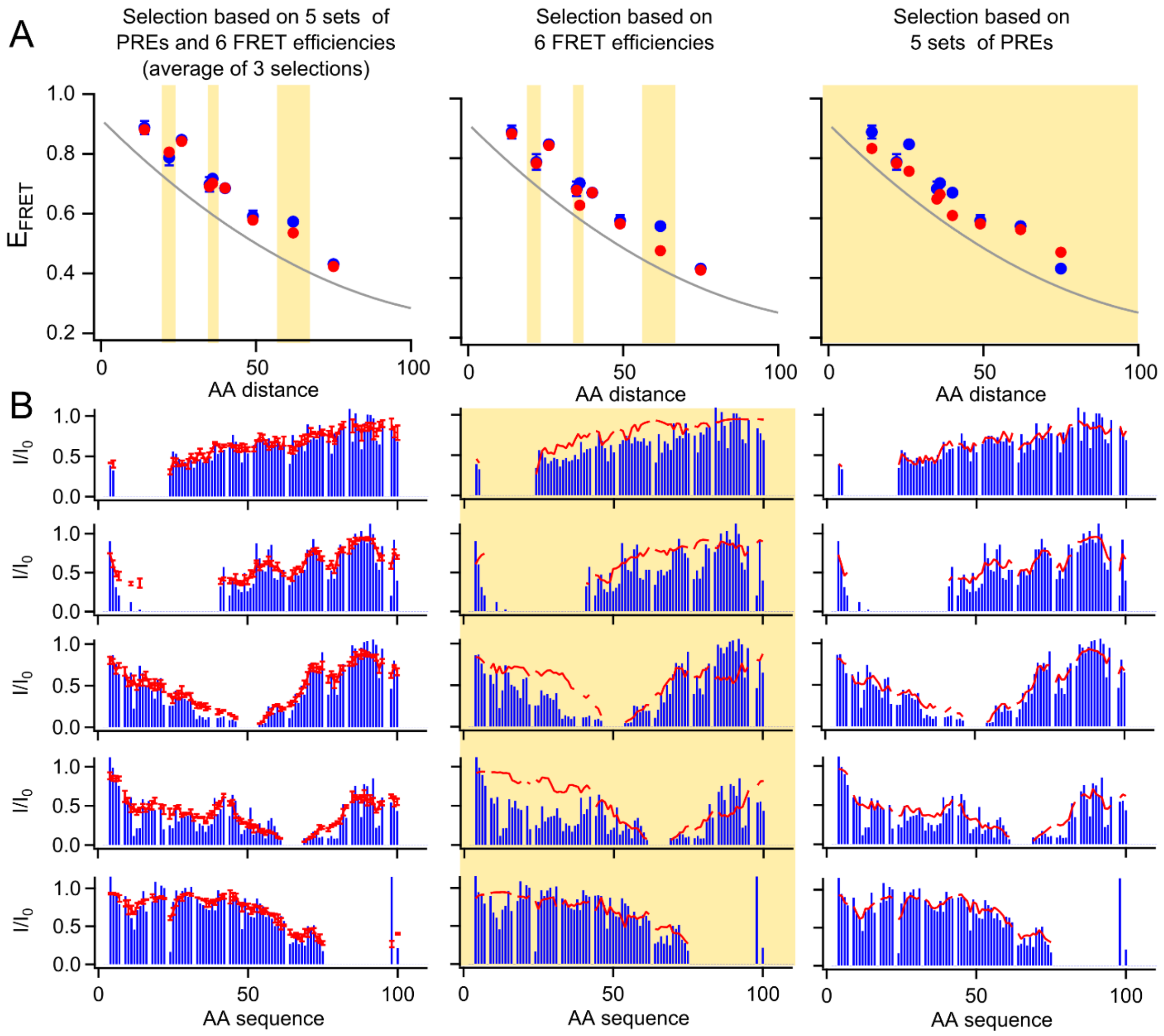

SI Figure 11: Complementarity of smFRET and NMR on the example of experimental data. (A) Experimental FRET efficiencies (E_{FRET}, blue points) with error bars corresponding to the standard deviation calculated from multiple experiments and E_{FRET} back-calculated from the selected conformational ensemble (red) plotted against the amino acid (AA) distance between the attached fluorophores. E_{FRET} expected from a flexible-meccano statistical coil ensemble is shown as a grey line. (B) Experimental PREs (blue) and PREs back-calculated from the selected conformational ensemble (red) are plotted as peak intensity ratios between the MTSL labelled and unlabeled protein (I/I_0) along the amino acid sequence. Left column: selection based on 5 sets of PREs and 6 FRET efficiencies; middle column: selection based on 6 FRET efficiencies, right column: selection based on 5 sets of PREs. Data not included in the selection and used for cross-validation are shown above yellow background. The selection based on FRET and PREs was performed three times to demonstrate reproducibility and its predictive character, also demonstrating sufficient sampling of the 200 conformer sized selected ensembles. The average FRET efficiencies and PREs are shown with their respective standard deviations across the three selections.

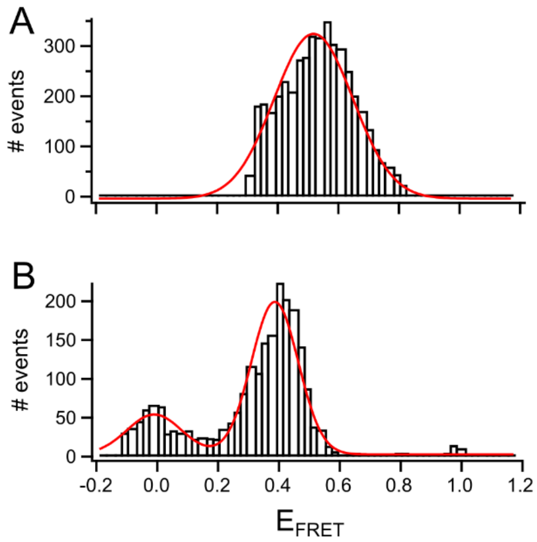

SI Figure 12: Validity of smFRET corrections and cross-validation. (A) smFRET histogram of $P_{1\text{-}100}$ C28 C64 labelled with Alexa488/Alexa647. The histogram originates from selecting specifically the FRET population in a 2D fluorescence lifetime versus FRET efficiency histogram accumulated over four one-hour long measurements. (B) smFRET histogram of sample '4-mid' from Hellenkamp et al., 2018,[5] labelled with Atto488/Atto594. Black bars represent experimental FRET histograms, red lines are double-Gaussian (B) or mono-Gaussian (A) fits from which FRET efficiencies were extracted.

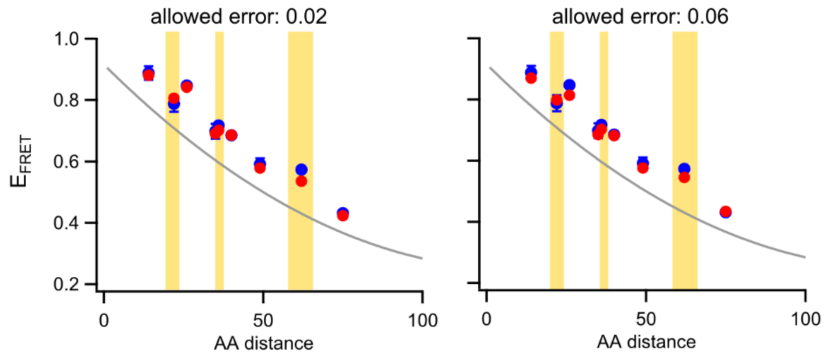

SI Figure 13: Comparison of E_{FRET} resulting from ASTEROIDS selection with allowed error of 0.02 (left) and 0.06 (right). Gray line: E_{FRET} expected from a statistical coil. Blue points: experimental E_{FRET} with standard deviation of repeated measurements as error. Red points: E_{FRET} calculated from the selected ensembles. On the left, the E_{FRET} calculated from three independent ASTEROIDS ensembles are averaged and standard deviations shown as errors.

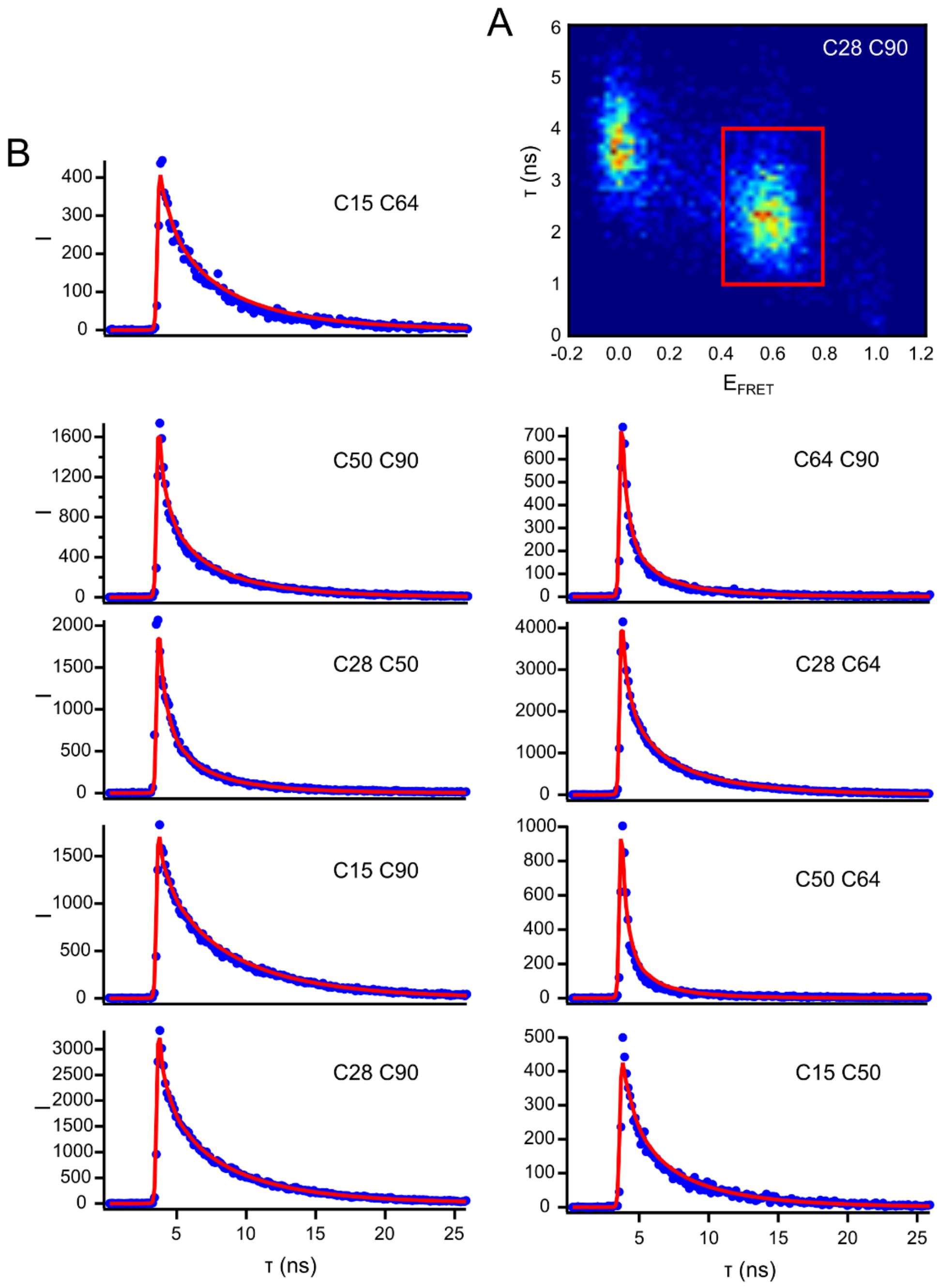

SI Figure 14: Description of fluorescence lifetimes by the selected ensemble. (A) Example 2D lifetime (τ) versus FRET efficiency (E_{FRET}) histogram. Red square denotes data for which lifetime histograms (B) were calculated. **(B)** Fluorescence lifetime histograms of the respective FRET populations (blue dots) and decay curves back-calculated from the selected ensemble (red) and including a scattering contribution. Scattering and the lifetime decay were scaled to best fit the experimental data.

labeling positions	AA distance	FRET efficiency			FRET efficiencies from ASTEROIDS selections					
		experimental	SD	# exp	FRET +PRE 1	FRET +PRE 2	FRET +PRE 3	FRET	PRE	FRET +PRE fast AV
C15 C64	49	0.59	0.02	2	0.58	0.58	0.58	0.58	0.58	0.58
C64 C90	26	0.85		1	0.84	0.84	0.84	0.84	0.76	0.85
C15 C90	75	0.43		1	0.42	0.42	0.42	0.43	0.49	0.42
C50 C90	40	0.69	0.01	2	0.68	0.69	0.68	0.69	0.61	0.71
C15 C50	35	0.70	0.02	7	0.69	0.69	0.69	0.69	0.66	0.70
C50 C64	14	0.89	0.02	2	0.88	0.88	0.88	0.88	0.83	0.88
C28 C50	22	0.79	0.03	2	0.80	0.80	0.81	0.78	0.78	0.76
C28 C64	36	0.72	0.01	2	0.70	0.70	0.70	0.64	0.68	0.65
C28 C90	62	0.57	0.01	2	0.54	0.53	0.54	0.49	0.56	0.49

SI Table 1: Labelling positions and FRET efficiencies of P_{1-100}. Labelling positions (positions of cysteines, C, in the amino acid chain), amino acid (AA) distance between the labels, experimentally measured FRET efficiencies averaged over the number of respective experiments (# exp) and the corresponding standard deviations (SD) are reported. FRET efficiencies from diverse ASTEROIDS selections are reported for the three independent selections using FRET and PREs, one selection using only FRET, one using only PREs and one using both FRET and PREs but under the assumption of fast averaging of the dye. Experimental FRET efficiencies displayed in red were used in the selection, those in black were kept for cross-validation. Note that chemical shifts were used in all selections reported.

labeling positions	r (Do)	r (Ac)
C15 C64	0.09	0.16
C64 C90	0.12	0.12
C15 C90	0.10	0.16
C50 C90	0.09	0.12
C15 C50	0.08	0.15
C50 C64	0.06	0.18
C28 C50	0.06	0.18
C28 C64	0.09	0.14
C28 C90	0.09	0.16

SI Table 2: Steady state fluorescence anisotropies of $P_{1\text{-}100}$. Anisotropies (r) were measured on the double labelled samples (Alexa488/Alexa594) and determined upon donor and acceptor excitation respectively on a PTI Quantamaster. A window of 5 nm around the emission peak was used to calculate r and a G factor determined at those wavelengths and under the same buffer conditions was used to correct the data. r was then determined as $r = \frac{I_{VV} - G \cdot I_{VH}}{I_{VV} + 2 \cdot G \cdot I_{VH}}$ with I_{VV} and I_{VH} being the fluorescence intensities measured with vertical poralized excitation and detection of vertical (parallel component) or horizontal emission (perpendicular component) respectively.

1. Walczewska-Szewc, K. & Corry, B. Accounting for dye diffusion and orientation when relating FRET measurements to distances: three simple computational methods. *Phys Chem Chem Phys* **16**, 12317–12326 (2014).
2. Jensen, M. R., Salmon, L., Nodet, G. & Blackledge, M. Defining conformational ensembles of intrinsically disordered and partially folded proteins directly from chemical shifts. *J. Am. Chem. Soc.* **132**, 1270–1272 (2010).
3. Nodet, G. *et al.* Quantitative description of backbone conformational sampling of unfolded proteins at amino acid resolution from NMR residual dipolar couplings. *J. Am. Chem. Soc.* **131**, 17908–17918 (2009).
4. Ozenne, V. *et al.* Flexible-meccano: a tool for the generation of explicit ensemble descriptions of intrinsically disordered proteins and their associated experimental observables. *Bioinformatics* **28**, 1463–1470 (2012).
5. Hellenkamp, B. *et al.* Precision and accuracy of single-molecule FRET measurements-a multi-laboratory benchmark study. *Nat. Methods* **15**, 669–676 (2018).
